# Abstracts from the 7th UK Congress on Obesity 2022

**DOI:** 10.1038/s41366-023-01306-4

**Published:** 2023-05-09

Lancaster University, Lancaster, 7^th^-8^th^ September 2022

**Sponsorship:** Publication of this supplement was sponsored by the Association for the Study of Obesity (ASO). All content was reviewed and approved by the ASO committee, which hold full responsibility for the abstract selections. The ASO is a registered charity (charity no. 1100648) and a UK company limited by guarantee (company no. 4796449). It is governed by an elected unpaid Board of Trustees, who meet up to three times a year.

## PROGRAM LISTING

### ASO SYMPOSIA

**7th September 2022**

**S01: Home**

**S02: Community**

**8**^**th**^ **September**

**S03: Retirement Village**

**S04: Schools**

### AWARD SESSION

**A01: 3-Minute Thesis Competition**

### ORAL SESSIONS

**7th September 2022**

**O1: Maternal and Child Obesity**

**8**^**th**^ **September**

**O2: Weight Management**

### POSTER SESSIONS

**7th & 8**^**th**^ **September 2022**

### ASO SYMPOSIA

## S01: Home

### S01 Newspaper media framing of maternal obesity in the UK: a review and framework synthesis

#### Heslehurst N^1^, Evans EH^2^, Incollingo Rodriguez AC^3^, Nagpal TS^4^ and Visram S^1^

##### ^1^Newcastle University, ^2^Durham University ^3^Worcester Polytechnic Institute, USA ^4^University of Alberta, Canada

Women are most likely to experience obesity discrimination, which is as prevalent as racism. During pregnancy, approximately two-thirds report experiencing weight-stigma. The news media is powerful at framing health issues such as obesity, and shapes public perceptions. This review explored UK newspaper portrayal of maternal obesity.

NexisUni was searched to identify national newspaper articles, published 01/2010-05/2021, reporting content on obesity during pregnancy. Articles were screened in full against the inclusion criteria. Framework synthesis integrated quantitative and qualitative analysis of the articles content.

Searches identified 442 articles that met the inclusion criteria: 59% published in tabloids, 41% in broadsheets. Overarching themes were the blame, responsibility, and burden of women”¯living with obesity. Women were directly blamed for their weight, pregnancy-related risks, and NHS care requirements. Solutions to maternal obesity were framed as being solely women’s responsibility to reduce their own (and future generations) weight, prevent adverse pregnancy outcomes, and alleviate the NHS burden. The burden of maternal obesity was ever-present: women were a burden on individuals (themselves, their children, health professionals), to society, and the NHS. Patterns in language framed the “problem and scale” of maternal obesity, emphasised risk, danger, and was alarmist, aggressive and violent. Articles platformed “experts” voices rather than women’s lived experiences. Throughout, these were underpinned by “oversimplifications” of obesity development, weight management strategies, and causal pathways to health outcomes.

This review identified that UK newspapers negatively frame maternal obesity. Maternal obesity is oversimplified: women are persistently blamed for having obesity, and its consequences, and are portrayed as solely responsible for solving maternal obesity. Exposure to blaming/alarmist messaging could increase stigma, women’s guilt relating to the potential for any adverse pregnancy outcomes and internalised weight bias. The power of newspaper media could, and should, be harnessed to de-stigmatise maternal obesity and promote maternal well-being.

Disclosures: None

### S02 The relationship between mental health and weight during the Covid-19 pandemic: A longitudinal study

#### Mueller J, Ahern AL, Sharp SJ, Davies A, Zuckerman A, Perry BI, Khandaker GM, De Lucia Rolfe E, Wareham N, Rennie K

##### University of Cambridge

Depression and anxiety have consistently been associated with obesity in observational studies. However, most studies make between-individual comparisons, which can mask important relationships. Less is known about within-individual associations. To address these limitations, we assessed longitudinal associations of depression, anxiety, and stress scores with self-reported body weight in a large sample at the within- and between-individual level during the COVID-19 pandemic.

Participants from a population-based cohort of UK adults completed monthly mental health and body weight measurements (August 2020 to April 2021). We used random intercepts regression models to examine longitudinal associations of depression, anxiety and stress with weight. In sub-group analyses, we included interaction terms of mental health variables with baseline characteristics (age, sex, BMI, education, occupation).

In 2133 participants, within-individual variation in depressive symptoms was associated with subsequent weight (0.045kg weight per unit of depression severity, 95% CI 0.021 to 0.069), but we did not find evidence for associations in between-individual analysis, or in analyses of stress and anxiety. In subgroup analyses, we found evidence of a dose-dependent moderation effect of baseline BMI on the within-individual association between depressive symptoms and subsequent weight (baseline BMI 25-29.9 kg/m2: 0.052kg weight per unit of depression severity, 95% CI 0.010-0.094kg; baseline BMI ≥ 30 kg/m2: 0.071kg weight per unit of depression severity, 95% CI 0.013-0.129kg). We did not find evidence for reverse causality.

In this study, individuals with overweight or obesity were more vulnerable to weight gain following higher-than-usual (for that individual) depressive symptoms than individuals with a healthy BMI. Although causality cannot be inferred, our analysis meets the Bradford-Hill criteria of biological gradient and temporality. Our results suggest that targeting depressive symptoms in individuals with overweight or obesity may aid weight management strategies.

Disclosures: None

## S02: Community

### S03 Calorie labelling on alcoholic beverages: attitudes, knowledge, and intentions to change behaviour

#### Sheen F, Brown J, Conway R, Esser S, Llewellyn C and Steptoe A

##### UCL

Introducing mandatory calorie labelling on alcoholic beverages is expected to result in individuals opting to consume fewer calories from alcoholic drinks, having the potential to impact obesity and increasing/higher risk alcohol consumption. There is moderate evidence that people are unaware of the energy content of alcoholic beverages, and support labelling to display this information. However, evidence for a beneficial effect on alcohol consumption is mixed, and some research indicates potential unintended consequences on health behaviours.

The current research examined the acceptance of labelling as a legal requirement, perceived usefulness of labelling, knowledge of calories in common alcoholic beverages, and intended changes in drinking. Relevant questions were added to the nationally representative Alcohol Toolkit Study of adults (18+) in England from November 2021 to January 2022.

There was strong support for calorie labelling on alcoholic beverages as a legal requirement from those who reported drinking alcohol (N = 3,716), with most (58.1%, 95%CI) agreeing that this information would be useful to them. This did not vary by AUDIT score (p = .170) or socioeconomic status (p = .197). Fewer than one in five (8.6-20.7%, 95%CI) accurately estimated the calorie content of servings of beer, wine, cider, or spirits, with most (36.4-56.7%, 95%CI) overestimating the calorie contents. In response to the prospect of calorie labelling on alcoholic beverages, only 15.9% (95%CI) indicated that they would decrease the number of beverages consumed at a typical drinking occasion. The majority (70.7%, 95%CI) suggested they would maintain their current number of beverages. However, of these individuals, a third indicated that they would perform at least one positive drinking behaviour change, with the most popular being to choose lower calorie alcoholic beverages.

Providing calorie information unambiguously on alcoholic beverages could improve knowledge among the majority of the English population, and may induce intentions to perform healthier drinking behaviours.

Disclosures: None

### S04 Type and density of food environment in an area of high deprivation and high childhood obesity: a geographical mapping study

#### Eskandari F, Lake AA and Butler M

##### Teesside University

The association between obesity and deprivation are strongly evidenced and socioeconomic disparities of exposure to the food environment are known to exist. Aims and objectives: Using Middlesbrough (in top 20% of the most deprived unitary authorities in England) case studies to understand, at micro level, what the food environment in an area of high deprivation and high childhood obesity looks like. Methods: The Food Standard Agency Food Hygiene Rating Scheme was used to provide data on food outlets. Percentage of obesity for children aged 4-5 and 10-11 years were obtained from the National Child Measurement Program at the ward level. Results: Mapping and overlaying of data indicated many of areas across Middlesbrough with high convenience and ‘instant’ food outlet concentration were also those where prevalence of child obesity and the level of deprivation were highest. Two wards that had the highest prevalence of childhood obesity for 10-11 year olds, also had the highest concentration of convenience and ‘instant’ food outlets. Within 400 m radius buffer distance from both primary and secondary schools, different types of takeaway outlets were accessible especially in the most deprived wards. Further geocoding indicated availability of fast-food outlets within 500 m, 800m and 1,000 m radius of both primary and secondary schools in different wards in which have the highest prevalence of childhood obesity and index of multiple deprivation. Conclusion: Overlaying of data allows to identify locations and populations which need the most attention from local government. While we have had successive obesity strategies, it highlights childhood obesity is an issue in the most deprived communities where the environment may not promote a healthy diet. Results of this study can be locally used as part of the evidence base in the formation of future policy regarding takeaway and fast-food outlets, especially in terms of school fringes.

Disclosures: None

## S03: Retirement Village

### S05 Exploring the experiences and motivations of adults living with excess weight regarding Weight Management Services (WMS): Findings from a cross-sectional survey conducted in Northern Ireland (NI)

#### Kyle E, Kelly, A, Woodside J, McGowan L

##### Queen’s University Belfast

Living with excess weight may negatively impact upon a person’s quality of life alongside increasing their risks for developing chronic illnesses. In Northern Ireland (NI), evidence-based weight management services (WMS) are limited. Understanding motivations and experiences of adults living with excess weight regarding engagement with WMS, is an important step in designing appropriate public health interventions.

A review of the literature and personal and public involvement and engagement (PPIE) shaped the development of an online, anonymous, cross-sectional questionnaire which ran between January-April 2022. It was advertised for NI-based adults aged 18 years plus, with experience of living with excess weight. It assessed previous experience with WMS and motivations for future WMS alongside socio-demographic characteristics. Opportunistic recruitment was used. Responses were analysed using descriptive statistics and significance testing as appropriate.

A total of n = 228 responses were eligible for analysis. All participants had personal experience of living with overweight or obesity, with 81% viewing themselves as currently living with excess weight. One fifth were male (20.6%) and 99.6% reported white Caucasian ethnicity. Most participants fell between 35-54 years old (43.4%) and 67.9% reported a university degree or higher. Almost all (94.7%) had tried to reduce their weight independently; 76% had not sought help from WMS. Regarding weight-loss attempts, 12.4% reported maintaining weight loss (for more than one year) with the majority regaining some, all or more weight (73%). The majority (92.4%) of participants who would like to reduce their weight have not sought support from WMS (p < 0.01). The top-rated motivation for accessing a WMS was improved health.

Whilst the majority of participants reported previous attempts to independently manage weight, engagement with WMS was limited in NI. Most respondents reported regaining weight lost and therefore future WMS focusing on health-gain (versus weight-loss) and broader motivations may be more appropriate.

Disclosures: None

### S06 The Effect of Exercise Training on Visceral Adipose Tissue in Adults: An Umbrella Review

#### Williams SRP, Herbert P, Petherick A, McKibben Mary-Ann

##### University of Wales Trinity Saint Davids

Visceral adipose tissue (VAT) is an important target for reducing the morbidity and mortality of obesity^1^. Evidence suggests that improvements in variables associated with cardiometabolic risk are related to VAT reduction independent of changes in subcutaneous adipose tissue^2^. As one of a series of evidence reviews commissioned by Public Health Wales to inform the National Exercise Referral Scheme (NERS), this umbrella review aimed to evaluate evidence of the effect of exercise training on VAT in adults.

Clinical practice guidelines and several databases were searched for systematic reviews, meta-analyses and pooled analyses of randomized and non-randomised controlled trials to March 2021. Any exercise intervention ≥1 week was included if change in absolute or relative VAT mass, volume or area was reported. Included reviews were appraised with the AMSTAR-2 critical appraisal tool^3^ and summarized narratively.

Eleven articles were included in total, 6 reviews were rated as ‘critically low’ and 5 as ‘low’ quality. Seven articles included meta-analysis and two meta-regression to estimate the dose-response relationship between exercise and VAT change. Among the main findings were: (i) all reviews reported reductions in VAT with aerobic exercise training (AEx) but, in comparison, resistance training has a weak or no effect; (ii) AEx of moderate-to-high intensity has a greater effect than low or high intensity; (iii) high-intensity interval training (HIIT) is effective for VAT reduction, but not more effective than continuous AEx; (iv) three reviews report AEx reduces VAT in the absence of weight loss, one reported AEx reduces VAT by a greater amount than a hypocaloric diet creating a similar energy deficit.

Review level evidence strongly supports the use of AEx of moderate-to-high intensity and HIIT for the reduction of VAT.

References

1. 10.3390/nu12040891

2. 10.1038/oby.2011.396

3. 10.1136/bmj.j4008

Disclosures: None

## S04: Schools

### S07 Evaluation of the Holiday Activities and Food Programme in Yorkshire

#### Gardner G, Sinclair M, Connolly A, Bryant M, Doherty B

##### University of York and The Food Foundation

The Holiday Activities and Food (HAF) programme aims to ease financial and food security pressures faced by families over school holidays by providing free meals and enriching activities to children eligible for benefits-related Free School Meals (FSMs). In 2020 the Government confirmed HAF would be expanded to all 151 top-tier authorities in England, over a minimum of six weeks in 2021.

An implementation evaluation was undertaken across four local authorities (LAs) in Yorkshire in the 2021 summer holidays. Data were gathered through a participatory ethnography of HAF activities (n = 104 hours); parent focus groups (n = 5); stakeholder interviews (n = 20); a nationally representative survey of children (n = 1418); and an analysis of local government documents.

Across the UK, 25% of children attended a summer holiday club (22% in Yorkshire). Food provision was deemed an essential element, and enriching activities encouraged children to be active, socialise and learn new skills e.g. cooking and crafts. Parents reported benefits, including the chance to work over holidays.

Relationships between schools, LAs and providers were instrumental in ensuring engagement with vulnerable families. Some areas offered places regardless of FSM eligibility, and there was no visible distinction between FSM/non-FSM children. As attendance was predominantly by children <12 years, LAs were keen that future provision would engage older children (e.g. through co-design).

The 2021 HAF programme was well received and ensured children received a healthy meal during school closure.

Our learning was shared with the Department of Education and escalated to support spending decisions. Key recommendations: (1) long-term funding of HAF is essential to continue building on foundations/partnerships between LAs and providers; (2) universal offering regardless of FSM eligibility may reduce stigma and continue to provide lifelines for families; (3) continued provision of varied, tasty meals should remain central to HAF, involving children/young people in planning.

Disclosures: None

### S08 The CONNECTS Food intervention; an implementation intervention to support primary schools adopt a whole school approach to food

#### Burton W, O’Kane N, Ahern S, Woodside J, Rutter H, Evans C, Spence S, Baker T, Sharif A and Bryant M

##### University of York

Schools provide the opportunity to promote healthy diets, reduce levels of obesity and inequalities through implementation of whole school approaches to food (e.g., food culture, environment, and education). Uptake of such approaches is often poor, partly because schools are complex systems with multiple competing demands. We co-designed an implementation intervention to support schools to identify and modify school food systems to promote system change.

We developed a systems map of primary school food with >100 stakeholders, including children, parents, teachers, headteachers and caterers. A co-design team used the Action Scales Model to guide development of the intervention across six online workshops which aimed to generate understanding of key principles for implementing the approach and identify the main barriers to adoption. We considered the feasibility of overcoming barriers, and the potential impacts of doing so, resulting in a priority list of actions. An action plan was then agreed, ensuring ‘points for action’ were included at multiple levels within the system (beliefs, goals, structures, events).

The CONNECTS-Food intervention aims to change the beliefs of system architects (e.g., head teachers) so that whole school approaches to food can be prioritised. It includes a web-based resource with recommendations, evidence of benefits, case studies and a tool to support schools develop their whole school approach to food strategy. An additional function is to reorient system goals by advocating policy change and encouraging key organisations (e.g., Ofsted) to monitor whole school approaches to food.

The CONNECTS-Food intervention has been developed to support schools to implement whole school approaches to food. It has received support from key organisations including DfE, keen to ensure as many children as possible benefit from a whole school approach to food. It is well placed to support strategies advocated in the recent UK Government Levelling up White paper.

Disclosures: None

### AWARD SESSIONS

## A01: ASO Prevention and Treatment Award 2022

### A01-01 Supporting Weight Management during COVID-19 (SWiM-C): a randomised controlled trial of an ACT-based intervention

#### Mueller J, Richards R, Jones RA, Whittle F, Woolston J, Stubbings M, Sharp SJ, Griffin SJ, Bostock J, Hughes CA, Hill AJ and Ahern AL

##### University of Cambridge

We evaluated the effect of a web-based, acceptance-based guided self-help intervention which aims to prevent weight gain in adults with overweight or obesity during the COVID-19 pandemic (SWiM-C: Supporting Weight Management during COVID-19).

We randomised 388 participants (≥18 years, BMI ≥ 25kg/m^2^) to the SWiM-C intervention (n = 192) or a control group (n = 196). SWiM-C is based on acceptance and commitment therapy (ACT) and is delivered remotely via an online web platform (12 weekly modules) and contact via telephone and email with a trained, non-specialist coach. The control group received a leaflet on weight management and wellbeing during the pandemic. Participants completed online questionnaires at baseline, 4 months, and 12 months. The primary outcome was change in self-reported weight from baseline to 12 months; secondary outcomes were eating behaviour, experiential avoidance, mental health, wellbeing and physical activity.

At 12 months, the adjusted difference in weight between the SWiM-C group and the control group participants was -0.81kg (95% confidence interval [CI]: -2.24 to 0.61kg). SWiM-C participants reported a greater reduction in experiential avoidance (-2.45, 95% CI: -4.75 to -0.15), uncontrolled eating (-3.36, 95% CI: -5.66 to -1.06), and emotional eating (-4.14, 95% CI: -7.25 to -1.02), and an increase in physical activity (8.96, 95% CI: 0.29 to 17.62) compared to the control group. No differences in mental health or wellbeing were observed at 12 months.

Whilst the effect of the SWiM-C intervention on weight was inconclusive, SWiM-C improved eating behaviours, physical activity and psychological flexibility. These variables have been previously identified as determinants of successful weight management. Further refinement of the intervention is necessary to ensure meaningful effects on weight prior to implementation in practice.

Disclosures: FW, JW, MS, SJS and JB report no conflicts of interest. AJH has consulted for Slimming World. CAH reports payment or honoraria from Ethicon, NovoNordisk and International Medical Press for lectures, presentations, speakers bureaus, manuscript writing or educational events. JM and RR are Trustees for the Association of the Study of Obesity (unpaid roles). ALA and SJG are the chief investigators on two publicly funded (MRC, NIHR) trials where the intervention is provided by WW (formerly Weight Watchers) at no cost outside the submitted work.

## A04: Best ECR Poster prize 2022

### A04-01 Development and initial evaluation of a weight management programme tailored for people with serious mental illness: a non-randomised feasibility study with qualitative interviews

#### Lee C, Piernas C, Waite F and Aveyard A

##### University of Oxford

People with serious mental illness (SMI) have higher rates of obesity and premature mortality due to cardiovascular disease (CVD) than the general population. Trials show behavioural weight management programmes (BWMPs) can help people with SMI lose weight and reduce the burden of CVD. However diagnostic-specific barriers to uptake and engagement are reported. We aimed to develop and evaluate a standard BWMP tailored for people with SMI – called ‘Weight cHange for people with sErious mEntal iLlness (WHEEL).’

The development comprised: 1) 12 patient and public contributors with SMI; 2) a systematic review of qualitative studies to identify programme characteristics that promote uptake and engagement for SMI; 3) a systematic review of trials testing BWMPs to identify which characteristics lead to weight loss; and 4) coding the effective characteristics against a standard 12-week BWMP to identify opportunities for tailored support. Initial evaluation comprised: 5) a non-randomised study of feasibility (retention and n, % of programme sessions attended) and acceptability (qualitative interviews plus self-reported weight loss) at end-of-programme.

The programme developed was a weekly BWMP delivered by a commercial company. It was augmented with a one-off educational session geared towards people with SMI and weekly mentor check-ins. Seventeen participants (mean age: 48·52 years; 47% with schizophrenia) enrolled in the feasibility study and 16 were followed-up at 12-weeks (95% retention). All participants attended the educational session, 9/16 attended 50% of the weekly BWMP sessions, and 12/16 responded to 50% of the weekly check-ins. All participants reported weight loss (mean 4·06kg, SD: 3·17) and valued the novel education and therapeutic support. However anxious avoidance remained a barrier to joining the BWMP.

This study showed initial evidence that a standard BWMP augmented with brief education and low-intensity support is feasible, acceptable, and may lead to weight loss in people with SMI.

Disclosures: None

## A05: 3-MINUTE THESIS COMPETITION

### A05-01 Changes in health-related quality of life and depressive symptoms in the first year following bariatric surgery: the BARI-LIFESTYLE observational study

#### Jassil FC, Carnemolla A, Kingett H, Doyle J, Lewis N, Montagut-Pino G, Kirk A, Marvasti P, Chaiyasoot K, Zakeri R, Mok J, Brown A, Elkalaawy M, Jenkinson A, Adamo M, Devalia K, Parmar C and Batterham RL

##### University College London

The desire to improve quality of life is one of the factors that motivates people to seek bariatric surgery. Currently, there is a paucity of prospective UK studies assessing the impact of bariatric surgery on health-related quality of life (HRQoL) and depressive symptoms.

Patients undergoing bariatric surgery at three NHS trusts were enrolled in the BARI-LIFESTYLE observational study and received post-bariatric standard care. HRQoL and depressive symptoms were assessed using EuroQol-5Dimensions-3Levels (EQ-5D-3L), Impact of Weight on Quality of Life-Lite (IWQoL-Lite) and Beck Depression Inventory-II (BDI-II) questionnaires at pre- and post-surgery (3-, 6- and 12-month). Anthropometric and objective physical activity data were also collected.

Prospective data from 77 patients (80.5% female) with a mean ± SD age of 43.4 ± 10.6 years and body mass index of 42.9 ± 5.8 kg/m^2^ were analysed. The EQ-5D-Index and EQ-Visual Analogue Score improved significantly post-surgery, mean (95% CI) improvement of 0.01 index (0.01 to 0.02) and 2.1% (1.7 to 2.5), both p < 0.001, respectively. Similarly, the post-surgery total IWQoL-Lite score increased significantly, 3.4% (2.9 to 3.8), p < 0.001, relative to the pre-surgery score. The BDI-II scores also showed significant improvement over time, -0.8 points (-0.9 to -0.6), p < 0.001. The improvement in HRQoL and depressive symptoms peaked in the first three months post-surgery. In multivariate linear regression, factors such as percentage weight loss (%WL), type of bariatric procedure and time spent on moderate-to-vigorous physical activity (MVPA) were found to mediate the improvement in HRQoL. At six months post-surgery, participants with no or minimal to mild depressive symptoms had significantly higher %WL compared to participants with moderate to severe depressive symptoms (21.3 ± 5.2 versus 15.6 ± 2.7%, p = 0.001).

Bariatric surgery improves HRQoL and depressive symptoms. The link between time spent on MVPA and HRQoL requires further investigation. Future studies should also elucidate the relationship between post-surgery depressive symptoms and weight loss outcomes.

Disclosures: None.

### A05-02 Protocol for the INFORMED (Individualised Patient Care and Treatment for Maternal Diabetes) study: A randomised controlled trial embedded within routine care

#### Dingena C, Mahendra A, Holmes M, Clements NS, Scott E and Zulyniak MA

##### University of Leeds

Women with pre-existing diabetes struggle to control glucose levels during pregnancy and are at high risk of pregnancy complications, compared to women without diabetes. Management of postprandial and nocturnal glucose levels are key targets to minimise risk of adverse events during pregnancy, yet women with pre-existing diabetes struggle to achieve optimal glucose levels. Therefore, the INFORMED trial aims to examine continuous glucose monitoring (CGM) glucose profiles across pregnancy affected by type 1- or type 2 diabetes (T1D/T2D) and how they are associated with personal, lifestyle characteristics and physiological parameters.

This double-blind randomised controlled trial aims to recruit pregnant women (n = 76) with pre-existing T1D/T2D at ~10-12 weeks’ gestation at NHS Leeds Teaching Hospitals. At each clinical visit across their pregnancy “’ 10-12, 18-20, and 28-34 weeks’ gestation “’ participants will consent to share medical records on general health, glycaemia using CGM, obstetric information, blood and urine sample for metabolomic analysis, and complete lifestyle and 24-hour diet questionnaires. Additionally, participants will consume two blinded experimental meals in duplicate at ~18-20 and ~28-34 weeks’ gestation. Primary outcomes include (i) effects of experimental meals on glycaemia and metabolism and (ii) association between metrics of glycemia and maternal and newborn health. Multiple variable analyses and clustering will be used to identify patterns and associations of interest.

The Leeds East Research Ethics Committee and National Health Service approved the study. We aim to publish our results in peer-reviewed scientific journals and disseminate our findings to the study participants and the wider public.

This study will contribute comprehensive and detailed records of shifts in maternal metabolism and glycemia throughout pregnancy in women with diabetes. Hopefully, providing insights to inform future precision therapies to manage maternal glycaemia and minimise maternal and offspring risks in women with diabetes in pregnancy.

Disclosures: None

### A05-03 The association between later evening meal consumption and body mass index (BMI), and psychological and behavioural characteristics of early versus late evening eaters in UK adults

#### Yan B, Caton S and Buckland N

##### University of Sheffield

Emerging evidence indicates the timing of food intake can be associated with BMI. However, findings are inconsistent and the psychological and behavioural characteristics of early versus late eaters are unclear. Therefore, this study aimed to i) assess the relationship between meal timing and BMI and ii) compare psychological and behavioural characteristics of early and late evening eaters. An online survey completed by 300 UK adults (analysed n = 260; 47% female; 37.46 ± 14.14 years; 26.18 ± 5.72 kg/m2) assessed meal timings (breakfast, lunch, evening meal and snacks), eating behaviour traits (uncontrolled eating, craving control, emotional eating, cognitive restraint, satiety responsiveness and intuitive eating) and health and weight management behaviours [e.g. physical activity, habitual diet (food frequency questionnaire) and sleep]. After ineligible participants were removed (n = 260), results showed that later evening consumption was not significantly associated with BMI (r = -0.09, p = 0.17). Hierarchical cluster analysis grouping participants as early or late eaters, showed that late eaters reported a significantly lower cognitive restraint (p < 0.05), longer daily consumption window (p < 0.001) and consumed high energy dense sweet snacks more frequently than early eaters (p < 0.01). There were no significant differences in BMI, craving control, uncontrolled eating, emotional eating, intuitive eating, satiety responsiveness, physical activity, sleep quality or sleep duration between early and late eaters. In this study, eating later was not associated with a higher BMI. However, late eaters may have less restraint over food intake and may be at increased risk of high energy dense sweet food intake and overconsumption. Further research is needed to explore the role of early and late food consumption for appetite control and BMI.

Disclosures: None

### A05-04 Experiences of obesity stigma, weight-based discrimination and views about people with obesity on the island of Ireland: findings from a mixed-methods study

#### Kelly A, McGowan L, Spyreli E, Heery E, Woodside JV, Croker H, Lawlor C, O’Neill R and Heinen MM

##### Queen’s University Belfast

Rising obesity levels over the last 50 years have been accompanied by an increase in negative attitudes towards people living with obesity (PwO), including stereotyping and bias. Weight bias/stigma can lead to weight-based discrimination.

Although research has documented experiences of weight stigma in countries with high obesity levels, research is lacking on the Island of Ireland (IoI). This study aimed to explore public views about PwO and capture perceived experiences of weight-based discrimination on IoI.

A nationally representative sample (n = 1049) from Northern Ireland/Republic of Ireland completed a telephone-based survey. Cross-sectional findings were supplemented with nine (n = 64) semi-structured online focus groups (FGs) in a sequential mixed-methods design.

Regression analyses examined survey respondents’ views about PwO and the prevalence of perceived weight-based discrimination (outcome variables). Univariate models tested the relationship between individual sociodemographic characteristics and outcome variables, whilst multivariable regression investigated the independent associations of age, gender, BMI, education and socio-economic status (SES) with the outcome variables.

One in ten survey respondents (n = 109) perceived that they had ever experienced weight-based discrimination. Of those that self-reported their height/weight and said yes to experiencing discrimination (n = 72), 89% had a BMI in the overweight/obesity category. Univariate regression models showed significant effects of BMI (p < 0.001), education (p = 0.019) and SES (p = 0.01) on perceived weight discrimination. When adjusted for confounders, the only significant association was BMI (p < 0.001). Although most had not personally experienced weight stigma/discrimination, the prevalent view within FG discussions was that it was common in society, despite confusion about what constitutes it. Positive attitudes towards PwO were generally observed, however views varied depending on demographics.

This was the first study on the IoI to explore public views on weight stigma, PwO and experiences of weight-based discrimination. FG discussions highlighted a need to increase public knowledge of weight stigma/discrimination and the complexity of obesity.

Disclosures: None

### A05-07 Study of how adiposity in pregnancy has an effect on outcome (SHAPES): a systematic review to identify datasets for an Individual Participant Data (IPD) meta-analysis

#### Nguyen G^1^, Vinogradov R^1 2^, Vale L^1^, Teare D^1^, Bigirumurame T^1^, Hayes L^1^, Lennie S^1^, Allotey J^3^ and Heslehurst N^1^

##### ^1^Newcastle University ^2^Newcastle upon Tyne Hospitals NHS Foundation Trust (NUTH) ^3^University of Birmingham

Maternal obesity increases the risks of adverse pregnancy outcomes. Extensive research with non-pregnant populations demonstrates that BMI poorly predicts risk compared with measures of body-fat/distribution (e.g. waist circumference). SHAPES is using risk prediction and health economics methods to identify measures of adiposity that could be used in early pregnancy to predict which women and babies would benefit most from additional care. This systematic review aims to identify existing datasets that could be used in an IPD risk prediction meta-analysis to explore adiposity in early pregnancy and pregnancy outcomes.

This systematic review is registered in PROSPERO (CRD42022310760). Searches include 6 databases (MEDLINE, EMBASE, PsycINFO, CINAHL (EBSCO), JBI Database of Systematic Reviews and Implementation Reports and Cochrane Library), international cohorts, reference lists, citations, and contacting authors. Screening has been carried out by two authors independently to identify datasets which contain variables for early pregnancy adiposity and pregnancy outcomes.

Searches resulted in 21,291 records after removing duplicates; 108 studies from 95 datasets were included. Sample sizes ranged from 29 to 22,223 women. Studies were conducted between 1984-2018. Study settings were Asia (n = 35), North America (n = 20), Europe (n = 23), Australia (n = 5), Africa (n = 4), South America (n = 7), North America (n = 20), and one multi-continent (Australia, New Zealand, the United Kingdom, and Ireland). Early pregnancy waist circumference was the most frequently reported adiposity measure, with the majority of outcome data related to gestational diabetes mellitus (~50%).

Authors of each eligible study will be invited to collaborate and provide their IPD data. Data sharing, collaboration and co-authorship agreements will be established. Each included dataset will be analysed individually, and meta-analysis methods will pool results across datasets. This research will add insight into current knowledge on whether adiposity measures are better than BMI at predicting risk, either on their own or combined with other measures.

Disclosures: None

### ORAL SESSIONS

## Maternal and Child Obesity

### O1 Midwives’ survey of their weight management practice before and after the GLOWING guideline implementation intervention: a pilot cluster randomised controlled trial (RCT)

#### Heslehurst N, McParlin C, Sniehotta FF, Rankin, J and McColl E

##### Newcastle University

Approximately 1 in 4 women enter pregnancy with an obese BMI. Maternal weight management interventions significantly improve maternal diet and physical activity behaviours, gestational weight gain, postnatal weight retention, and some pregnancy outcomes. UK pregnancy weight management guidelines were published in 2010; however, low self-efficacy is a core barrier to midwives’ implementation. GLOWING used social cognitive theory (SCT) to address evidence-based barriers to practice with the aim of supporting midwives’ guidelines implementation.

A pilot cluster RCT in four NHS Trusts (clusters) in a deprived region of England. Clusters were randomised to intervention (midwives received the intervention) or control (no intervention). One-hundred midwives were randomised to complete questionnaires pre-/post-intervention. Guideline recommendations for midwives’ practice were grouped into categories: 1) communication-related behaviours (including weight- and risk-communication); 2) support/intervention-related behaviours (including diet/nutrition, physical activity, weight management, referrals/signposting). Questionnaires used SCT constructs (self-efficacy, outcome expectancies, intentions, behaviours) and a 7-point Likert scale converted to a 0-100 scale. Higher scores were more positive. Descriptive statistics were used to compare SCT constructs between intervention and control arms, pre-/post-intervention.

74 midwives consented, 68 returned questionnaires. Pre-intervention, self-efficacy for support/intervention-related behaviours scored lowest. In controls, there was limited difference between pre-/post-intervention scores. Post-intervention, mean [SD] scores were consistently higher among intervention midwives than controls, particularly for support/intervention self-efficacy (71.4 [17.1] vs. 58.4 [20.1]). Self-efficacy was higher post-intervention than pre-intervention for all outcomes among intervention midwives: weight-communication (76.3 [16.7] vs. 67.2 [21.1]), risk-communication (79.4 [16.4] vs. 68.6 [14.9]), diet/nutrition/physical activity (76.4 [16.0] vs. 49.3 [16.5]), weight management (72.1 [18.3] vs. 48.3 [19.8]), referrals/signposting (63.3 [26.0] vs. 47.9 [17.3]), and consistently higher than controls.

Results support the theoretical models used to develop GLOWING: low self-efficacy is a core implementation barrier. The pilot results suggest GLOWING successfully targets self-efficacy, potentially with a positive impact on guideline implementation.

Disclosures: None

### O02 The associations between weight status and mental health outcomes before, during and after pregnancy

#### Griffiths A, Shannon O, Brown T, Jones A, Swann C, Davison M, Ells L and Matu J

##### Leeds Beckett University

Women living with obesity around pregnancy have been reported to have an elevated risk of adverse mental health outcomes (Bogaerts et al. 2013). Nevertheless, these associations have not been observed in all studies (Insan et al. 2020), which could be related to differences in study design or participant characteristics. As such, this meta-analysis aimed to explore the associations between weight status and mental health outcomes in women living with obesity around pregnancy.

MEDLINE, PsycINFO and Embase were searched from 1st January 2000 until 15th July 2021. Studies were prospective cohort and longitudinal of any duration which aimed to assess the association between excess weight and mental health outcomes in women pre (up to 3-months prior to conception), during or post-pregnancy (up to 12-months after pregnancy). Data were analysed via narrative synthesis and random effects meta-analyses.

Scores on mental health indices were significantly greater (indicative of worse mental health) in individuals with obesity compared to individuals of a healthy weight (SMD = 0.21 [95% CI: 0.11 to 0.31], I2 = 73%, p < 0.01). There was a small increase in depressive symptoms in individuals with obesity compared to those of healthy weight (SMD = 0.23 [95% CI: 0.13 to 0.34], I2 = 75.0%, p < 0.01). There was a small increase in trait anxiety symptoms in women living with obesity compared to those of healthy weight (SMD = .24 [95% CI: .01 to .47], I2 = 83.7%, p = .039 and no significant differences regarding state anxiety symptoms. Narrative evidence suggests that socioeconomic status and ethnicity may modify the relationship between obesity and mental health symptomology.

The findings of this meta-analysis indicate that maternal obesity is associated with adverse mental health. These findings may be used to inform the design of maternal weight management interventions and programmes.

Disclosures: None

### O03 Cultural adaptation of a lifestyle and weight management text message intervention for women in the postpartum period

#### Spyreli E^1^, Caperon L^2^, Ansell E^2^, Ahern S^2^, Bridges S^2^, Dombrowski SU^3^, Hoddinott P^4^, Free C^5^, Coulman E^6^, Anderson AS^7^, McIntosh E^8^, McDowell C^9^, Kee F^1^, Woodside JV^1^, Cardwell C^1^, Gallagher D^1^ and McKinley MC^1^

##### ^1^Queen’s University Belfast ^2^Bradford Institute for Health Research, ^3^University of New Brunswick, ^4^University of Stirling,^5^London School of Hygiene & Tropical Medicine, London, ^6^University of Cardiff, ^7^University of Dundee

The library of text messages for the Supporting MumS (SMS) intervention was developed with extensive patient and public involvement (PPI) to help with postpartum weight management; a successful feasibility study was conducted in Northern Ireland (https://www.journalslibrary.nihr.ac.uk/phr/phr08040/#/abstract). This work aimed to conduct further PPI with postpartum women who have struggled with their weight to review and adapt the text message library to ensure it is acceptable and culturally relevant in preparation for a UK-wide trial.

PPI partners were recruited from Scotland and England, via existing community networks, with a focus on reaching mothers from non-White ethnic backgrounds. Initial online group PPI discussions were conducted to introduce the SMS intervention and review a selection of text messages in relation to their language, humour, clarity, tone and cultural relevance. This was followed by one-to-one PPI; mums were sent the library of text messages, via post or email, and invited to review and provide feedback. This was an iterative process.

The text message library adaptation (n = 357) took nine months. Nineteen women responded to the PPI invitation: n = 4 London (African-Caribbean), n = 8 Bradford (Asian), n = 7 Scotland (white). Twelve joined the online discussions [London (n = 4), Bradford (n = 8) and 12 provided individual feedback on the text message library (n = 3 London, n = 3 Bradford, n = 6 Scotland).

Mums commented positively on the friendly, supportive, encouraging and empathetic tone of the messages. Adaptations suggested were minor and included: removing idioms and colloquialisms that might not be widely understood (e.g. ‘take the bull by the horns’); being more concise; and including more evidence-based web links.

PPI directed the cultural and regional adaptation of the text message library. The effectiveness and cost-effectiveness of the Supporting MumS intervention will be tested in a UK-wide trial (https://fundingawards.nihr.ac.uk/award/NIHR131509).

Disclosures: None

### O04 Willingness-to-taste vegetables and fruit may be context dependent in 4-7 year old children

#### Wilkinson NM^1^, Kannan S^2^, Ganguli H^3^, Hetherington MH^1^ and Evans CEL^1^

##### ^1^University of Leeds, ^2^University of Michigan ^3^University of Cumberland Kentucky

We are conducting a repeated-measures randomised controlled trial to determine effects of sensory food education on 4-7 year olds’ willingness-to-taste (WTT) fruit and vegetables (FV). WTT is one aspect of food fussiness and neophobia, which can contribute to low FV consumption in children. We expected that baseline WTT would align with food fussiness ratings from caregivers, and that reluctance to taste would be common.

Children in participating classes took part in a simple, fun tasting activity in which they could taste as many of nine FV samples as they wished. WTT is the total number of food samples tasted out of nine. Caregivers who used the online consent route completed a subset of the validated Child Eating Behaviour Questionnaire (CEBQ).

In total, 59% of n = 329 children (194) tasted all nine samples. Only 13% of children tasted four or fewer of the samples. 5% tasted none of the samples. The mean number of samples tasted was 7.43 out of 9 (SD 3). Out of 55 children rated, the seven children rated most food-fussy and neophobic on the CEBQ scale by caregivers averaged a WTT of 7.57. Thus, measured WTT did not consistently follow caregiver ratings. WTT in education/play food tasting activities may be a different thing to WTT at mealtimes.

The high WTT before any intervention, including for many ‘food-fussy/neophobic’ children, suggests that simply providing contextually conducive opportunities to taste FV could avoid some barriers to tasting and hence support many children to expand the variety of FV they know and like. It remains to ascertain which aspects of the tasting activity were successful in engaging children; we hypothesise that (a) separating tasting activities from mealtimes, (b) validating both liking and disliking, (c) simple gamification, (d) a small peer group, will all encourage tasting.

Disclosures: None.

### O05 Prospective associations between parental feeding practices in early childhood and the onset of eating disorder symptoms in adolescence using Generation R and The Gemini Study

#### Kininmonth AR, Harris HA, Nas Z, Derks I, Boniface D, Jansen PW and Llewelyn CH

##### University College London

Nonresponsive parental feeding practices (PFPs) that are controlling or coercive (e.g., pressure, restriction, instrumental feeding) are thought to interfere with a child’s ability to self-regulate their food intake and are associated with variation in appetite avidity and adiposity. We hypothesise that nonresponsive PFPs that undermine a child’s autonomy of food intake may also predispose to eating disorders (EDs) that directly influence weight status (e.g. Binge Eating Disorder). However, virtually nothing is known about the relationship between PFPs and the onset of ED symptoms in adolescence.

Data were from two population-based cohorts with harmonised measures of PFPs and ED symptoms: Gemini (n = 876) and Generation R (GenR; n = 2825). Parents self-reported pressure to eat, restriction and instrumental feeding when children were 4-5 years old. Adolescents self-reported compensatory behaviours (e.g. meal-skipping, purging), restrained eating, binge eating, uncontrolled eating and emotional eating at 12-14 years. Ordinal/binary logistic regression analyses were performed (categorical DVs: ED symptoms; continuous IVs: PFPs), adjusting for clustering within families (Gemini), sex, ethnicity, age, income, maternal education, gestational age and child BMIz.

In Gemini, greater pressure to eat in early childhood increased the odds of adolescents engaging in compensatory behaviours (OR;95% CI = 1.27;1.12-1.45; p < 0.001). In GenR, greater parental restrictive feeding was associated with higher odds of compensatory behaviours (OR = 1.12;1.04-1.20; p < 0.01), restrained eating (OR = 1.12;1.04-1.20; p < 0.01), uncontrolled eating (OR = 1.14;1.06-1.22; p < 0.001) and emotional eating (OR = 1.15;1.05-1.26; p = 0.003) in adolescence. Greater instrumental feeding was associated with higher odds of binge eating in GenR (OR = 1.15;1.03-1.29; p = 0.01) and uncontrolled eating in Gemini (OR = 1.16;1.02-1.32; p = 0.026).

This is the first study to explore if obesity-associated PFPs are also risk factors for ED symptoms. This novel examination suggests that greater exposure to nonresponsive PFPs in early childhood are associated with an increased risk of ED symptoms in adolescence, alongside other established outcomes including appetite avidity and obesity. Findings, however, were inconsistent across cohorts and need replicating.

Disclosures: None

### O06 Obesity-related appetitive traits as predisposing risk factors for binge-eating symptoms in early adolescence: results from the Gemini Twin study and the Generation R study

#### Nas Z, Derks I, Kininmonth AR, Harris HA, Jansen PW and Llewelyn CH

##### University College London

Individual differences in appetitive traits (ATs) (such as food cue responsiveness and emotional eating) emerge in early life, are moderately stable across childhood, and are associated with obesity risk. Obesity and Binge-Eating Disorder (BED) are closely linked, yet little is known about the extent to which early appetite also predisposes to BED. We used data from two prospective cohorts with harmonized measures to examine this association.

Data were from the Generation R Study (GenR, n = 2801) and the Gemini twin cohort (Gem, n = 869). ATs were measured using the parent-report Child Eating Behaviour Questionnaire at 4 (GenR) and 5 years (Gem). DSM-V classifiable subclinical binge eating (BE) symptoms (i.e. overeating and loss of control eating) were self-reported by children at 12-13 (Gem) and 14 years (GenR) using the Development And Well-being Assessment (DAWBA). Ordinal logistic regression analyses were performed for each AT (7 continuous IVs) with BE symptoms (binary DV: 0/ 1 symptom presentation), adjusting for clustering of twins in families (Gem) and covariates (sex, ethnicity, age, income, maternal education, gestational age, child BMI).

In both cohorts, greater food responsiveness was significantly associated with higher odds of BE (Gem: OR = 1.71, 95%CI = 1.25, 2.33, GenR: OR = 1.41, 95%CI = 1.20, 1.65). In Gemini only, greater enjoyment of food (OR = 1.61, 95%CI = 1.06, 2.45) and slower eating (OR = 0.66, 95%CI = 0.47, 0.95) were also significantly associated with higher and lower odds of BE respectively.

Greater food cue responsiveness in early childhood may be a predisposing risk factor for BE symptoms in early adolescence, alongside susceptibility to obesity. Greater enjoyment of food and more rapid eating in early childhood may also be implicated, although these findings were inconsistent across cohorts and require replication. These results implicate appetite avidity as a common aetiological risk factor for both obesity and BE symptoms, that is observable and measurable in early life.

Disclosures: None

## O2: Weight Management

### O07 A natural experiment comparing the effectiveness of the “Healthy Eagles” child weight management intervention in school versus community settings

#### Little M, Serber-Souza S, Kebbe M, Spratt T, Aveyard P, Jebb SA

##### University of Oxford

Behavioural weight management interventions are recommended for the treatment of obesity in children. However, the evidence for these is limited and often generated under trial conditions with White, middle-class populations. Healthy Eagles is a behavioural weight management intervention designed to treat excess weight in children. It ran in the London Borough of Croydon from 2017 to 2020 and was delivered in both school and community settings, providing a natural experiment to compare outcomes. A total of 1560 participants started the Healthy Eagles programme; 347 in the community setting and 703 in the school setting. Data were analysed for those who completed 70% of the programme. In the school setting, there was a small but significant reduction in BMI z-score (M = -0.04, 95% CI = -0.08, -0.01) for participants above a healthy weight, especially in those with severe obesity (M = -0.09, 95%CI = -0.15, -0.03); there was no significant change in any subgroup in the community setting. Linear regression analysis showed the school setting was associated with a 0.26 (95%CI = 0.13, 0.49) greater reduction in BMI Z-score than the community setting after adjusting for ethnicity, deprivation, age and gender. Across both programmes, the effect was somewhat greater in participants from a Black (African/Caribbean/Other) ethnic background (M = -0.06 95%CI = -0.09, -0.02) and from the two most deprived quintiles (M = -0.06, 95%CI = -0.11, -0.01). Data were limited, but minimal changes were measured in nutrition and physical activity behaviours regardless of setting. This evaluation provides indirect evidence of a small but significant benefit to running weight management interventions in a school versus community setting.

Disclosures: M.L. and S.S.S. are the directors of Foodtalk CIC and therefore earned a personal wage for running the Healthy Eagles programme.

### O8 BEhavioural Weight Management: COMponents of Effectiveness (BE:COME): Descriptive data findings

#### Gregg R, Sharif S, Avenell A, Ells L, Jaiswal N, Jayacodi S, MacKenzie R, Simpson S, Wu O, Logue J

##### Lancaster University, The University of Glasgow, Leeds Beckett University, The University of Aberdeen

Behavioural weight management interventions (BWMIs) are the main funded interventions for obesity in the UK, but current services vary considerably. Comparative research is hampered by the lack of detailed intervention descriptions, standardised outcome measures, and published studies that reflect these services. This serves as a major barrier for commissioners of these vital services leading to the current situation of variable funding and provision across the UK. The research aims to determine which individual components of BWMIs are associated with greater attendance, intervention completion and weight loss, and compare them using network meta-analysis at intervention level and component network meta-analysis.

To map individual components of behavioural weight management interventions used in pragmatic clinical trials and those commissioned in the real-world. To use an independent advisory group to define components and behavioural change techniques hypothesised to be of importance.

Intervention descriptions were collected via a standardised intervention description template (STARLITE) (Heggie, Mackenzie et al. 2020). STARLITE was transferred into Qualtrics software, version July 2021, copyright 2020 Qualtrics, and distributed via Qualtrics link. The RCT templates were completed by the researcher using research protocols and study materials. An independent advisory group decided on the components of importance in the analysis. Descriptive methods were used to summarise the data in each component.

12 components/co-variables were chosen for analysis, these include (but not limited to) participant referral, pre-assessment, delivery and monitoring modes, tailoring and adaptation, weighing and staffing. Differences were seen in many of these components such as service interaction with referrers, conduct of pre-assessment, delivery in groups or individually, tailoring to a specific group and staff continuity.

This research has confirmed the complexity of BWMI and the need for standardised reporting. Using novel approaches this research will compare within these specific components to identify effective and cost-effective approaches.

Disclosures: None

### O9 Changes in health-related quality of life and depressive symptoms in the first year following bariatric surgery: the BARI-LIFESTYLE observational study

#### Jassil FC, Carnemolla A, Kingett H, Doyle J, Lewis N, Montagut-Pino G, Kirk A, Marvasti P, Chaiyasoot K, Zakeri R, Mok J, Brown A, Elkalaawy M, Jenkinson A, Adamo M, Devalia K, Parmar C and Batterham RL

##### University College London

The desire to improve quality of life is one of the factors that motivates people to seek bariatric surgery. Currently, there is a paucity of prospective UK studies assessing the impact of bariatric surgery on health-related quality of life (HRQoL) and depressive symptoms.

Patients undergoing bariatric surgery at three NHS trusts were enrolled in the BARI-LIFESTYLE observational study and received post-bariatric standard care. HRQoL and depressive symptoms were assessed using EuroQol-5Dimensions-3Levels (EQ-5D-3L), Impact of Weight on Quality of Life-Lite (IWQoL-Lite) and Beck Depression Inventory-II (BDI-II) questionnaires at pre- and post-surgery (3-, 6- and 12-month). Anthropometric and objective physical activity data were also collected.

Prospective data from 77 patients (80.5% female) with a mean ± SD age of 43.4 ± 10.6 years and body mass index of 42.9 ± 5.8 kg/m^2^ were analysed. The EQ-5D-Index and EQ-Visual Analogue Score improved significantly post-surgery, mean (95% CI) improvement of 0.01 index (0.01 to 0.02) and 2.1% (1.7 to 2.5), both p < 0.001, respectively. Similarly, the post-surgery total IWQoL-Lite score increased significantly, 3.4% (2.9 to 3.8), p < 0.001, relative to the pre-surgery score. The BDI-II scores also showed significant improvement over time, -0.8 points (-0.9 to -0.6), p < 0.001. The improvement in HRQoL and depressive symptoms peaked in the first three months post-surgery. In multivariate linear regression, factors such as percentage weight loss (%WL), type of bariatric procedure and time spent on moderate-to-vigorous physical activity (MVPA) were found to mediate the improvement in HRQoL. At six months post-surgery, participants with no or minimal to mild depressive symptoms had significantly higher %WL compared to participants with moderate to severe depressive symptoms (21.3 ± 5.2 versus 15.6 ± 2.7%, p = 0.001).

Bariatric surgery improves HRQoL and depressive symptoms. The link between time spent on MVPA and HRQoL requires further investigation. Future studies should also elucidate the relationship between post-surgery depressive symptoms and weight loss outcomes.

Disclosures: None.

### O10 Promising evidence that a low-energy total diet replacement programme is safe and efficacious for the treatment of non-alcoholic steatohepatitis

#### Koutoukidis DA, Jebb SA, Mozes FE, Tomlinson JW, Pavlides M, Saffioti F, Aveyard P, Cobbold JF

##### University of Oxford

Although low-energy diets are used to treat obesity and type 2 diabetes, concerns exist that they may worsen the liver disease in patients with non-alcoholic steatohepatitis (NASH) and moderate to advanced fibrosis. We aimed to test the safety and efficacy of a low-energy total diet replacement programme in this population.

In this single-centre, 24-week, single-arm trial (ISRCTN12900952), 16 adults with body mass index (BMI) ≥ 30kg/m^2^ and biopsy-proven NASH with fibrosis stage 2-3 received remote dietetic support to follow a low-energy (~880kcal/day) total diet replacement programme for 12 weeks and stepped food reintroduction for another 12 weeks. Trained assessors blindly evaluated markers of liver fat [magnetic resonance imaging proton density fat fraction (MRI-PDFF)], fibro-inflammation (corrected T1), and fibrosis [liver stiffness on vibration-controlled transient elastography (VCTE)]. Safety signals included adverse events and liver biochemical markers.

Results: The median (IQR) BMI was 36.2kg/m^2^ (34.2-39.1) and six participants had type 2 diabetes. Fourteen participants provided data at 24 weeks (n = 13 for MRI data). Weight loss at 24 weeks was 15.0% (95%CI: 11.2%-18.6%, p < 0.001). Compared with baseline, MRI-PDFF reduced by 76% (95%CI: 55%-84%, p < 0.001), corrected T1 by 17.1% (95%CI: 11.6%-23.0%, p < 0.001, and VCTE-estimated liver stiffness by 41.6% (95%CI: 31.2%-51.4%, p < 0.001). No participants showed worsening of the markers. The proportion of patients achieving normalisation based on established clinical cut-offs for MRI-PDFF (<5.6%), corrected T1 (<795ms), and VCTE-estimated liver stiffness (<6.5kPa) were 69%, 50%, and 79%, respectively. There were no serious adverse events related to the intervention. Mild transient increases in liver biochemical markers at week 4 in one patient resolved by week 24.

The low-energy total diet replacement programme is highly adhered to and shows good safety profile and promising efficacy in the treatment of NASH. A definitive trial is needed to assess whether it reverses the natural history of the disease trajectory.

Disclosures: DAK, SAJ, PA, JWT, MP and JFC are investigators in another investigator-initiated NIHR-funded trial where the intervention is donated to the University of Oxford by Nestle Health Science and Oviva outside the submitted work. MP is a shareholder in Perspectum.

### O11 Two-year Effect of Semaglutide 2.4 mg on Control of Eating in Adults with Overweight/Obesity: STEP-5

#### Abbott S, Wharton S, Batterham RL, Bhatta M, Buscemi S, Christensen LN, Frias JP, Jódar E, Kandler K, Rigas G, Wadden TA, Garvey WT

##### Coventry University, York University, McMaster University Canada, Wharton Weight Management Clinic, University College London

STEP-5 (NCT03693430) investigated once-weekly (OW) subcutaneous semaglutide 2.4mg versus placebo for overweight/obesity treatment in adults over 2 years.

Adults with body mass index (BMI) ≥ 30kg/m2, or ≥27kg/m2 and ≥1 weight-related comorbidity, without diabetes, were randomised 1:1 to semaglutide 2.4mg OW or placebo for 104 weeks. Co-primary endpoints were body weight (BW) changes. Control of eating (CoE) questionnaire (CoEQ) was assessed in a subgroup from Canada/USA, with scores from 19 individual items grouped into 4 domains: craving control, craving for savoury, craving for sweet, or positive mood. P-values for exploratory CoEQ data were unadjusted for multiplicity.

304 adults were randomised (78% female; mean age 47 years, BW 106.0kg and BMI 38.5kg/m2). Semaglutide significantly reduced BW from baseline to week 104 versus placebo (estimated treatment difference: −12.6 %-points [95%CI: –15.3, –9.8]; p < 0.0001). In participants completing the CoEQ with semaglutide (n = 88) versus placebo (n = 86), all 4 domain scores significantly improved at week 20 and 52 (all p < 0.05). At week 104, craving control and craving for savoury domains remained significantly improved with semaglutide versus placebo (p < 0.01); positive mood and craving for sweet were not significant. Scores for the following craving-related items were significantly reduced with semaglutide versus placebo at week 104: desire to eat salty and spicy food, craving for dairy food, craving for starchy food, difficulty in resisting cravings, and difficulty in CoE (all p < 0.05). Scores for hunger and fullness improved with semaglutide versus placebo at week 20, 52 and 104, but the differences were only significant at week 20 (both p < 0.001).

In adults with overweight/obesity, substantial weight loss with semaglutide 2.4mg was accompanied by short- and long-term improvements in CoE vs placebo, with the greatest effect on craving control and craving for savoury foods.

Disclosures: SA: Speaker honorarium from Johnson & Johnson; non-paid committee roles: British Obesity and Metabolic Surgery Society, British Dietetic Association (BDA) Obesity Specialist Group, Obesity Management Collaborative UK; funded grants: publication grant from

### O12 Patient experiences of accessing specialist weight management services - Are there lessons to be learnt on how best to standardise services and optimise outcomes?

#### Swancutt D, Watkins R, Alexander M, Lloyd J, Burns L, Tarrant M, Pinkney J

##### University of Plymouth

Accessing specialist weight management services can be a daunting prospect for people who have severe obesity, with psychological and logistical obstacles to overcome just to attend their first appointment. Many specialist services have instigated changes over time to improve access. Yet, for some individuals a disjoint still remains in their experiences of this.

This study aimed to understand how services have developed their patient pathways over time and how current access to services is experienced by patients.

The study involved qualitative analysis of national online patient forums and interviews with patients, healthcare staff and commissioners in South West England (N = 24). Anonymised data was extracted from 57 relevant threads containing 4,832 online patient forums posts. Framework analysis was applied to code, chart and analyse findings.

The themes in patient experience related to: perception of variability of services and waiting times; the challenge of access for those with co-morbidities; perceived stigma in communication around weight change through referral routes; reassurance of receiving peer support; and the importance of patient-relevant, rather than biochemical, health outcomes.

In addressing access issues, key local service developments included: initiating early and ongoing engagement with patients coming into the service to alleviate frustration and anxiety; using ‘buddy systems’ to help patients settle into treatment groups. Finally, once established, remote delivery was believed to require fewer resources and prompt greater attendance in part because of the convenience this represented for patients.

Across the UK there remains some anxiety and confusion amongst patients when accessing weight management services, primarily around service availability, waiting times and perceived stigma. Local service improvements enhance access, though are not universally available.

Despite limited evidence of effectiveness, patients reported a preference for the convenience of remote delivery, which also offers potential resource savings for services.

Disclosures: None

## POSTER SESSIONS

### P01 The efficacy of GLP-1 receptor agonists for the management of postprandial hyperinsulinaemic hypoglycaemia following bariatric surgery: A systematic review and narrative synthesis

#### Llewellyn D, Logan Ellis H, Aylwin S, Oštarijaš E, Green S, Sheridan W, le Roux C, Miras A, Patel A, Vincent R, Dimitriadis G

##### King’s College Hospital NHS Foundation Trust

Postprandial hyperinsulinaemic hypoglycaemia with neuroglycopenia is an increasingly recognised complication of Roux-en-Y gastric bypass (RYGB) and gastric sleeve surgery and may detrimentally affect patient quality of life. One likely causal factor is gut hormone Glucagon Like Peptide-1 (GLP-1), which has an exagerated rise following ingestion of carbohydrates in patients who have had bariatric surgery. We sought to assess the role of GLP-1RA in managing postprandial hypoglycaemia following bariatric surgery.

Medical Literature Analysis and Retrieval System Online (MEDLINE), Excerpta Medica (EMBASE), Cochrane Central Register of Controlled Trials (CENTRAL), ClinicalTrials.gov, and SCOPUS databases were systematically and critically appraised for all peer reviewed publications that suitably fulfilled the inclusion criteria established a priori. The protocol for this systematic review was developed according to the Preferred Reporting Items for Systematic review and Meta-Analysis Protocols. It followed methods outlined in The Cochrane Handbook for Systematic Reviews of Interventions and is registered with PROSPERO (ID CRD420212716429).

Our search produced only 6 articles that met the criteria for inclusion into this qualitative synthesis. Of the published articles, there was a cohort study of 5 patients, two case reports and 3 randomised controlled trials (RCTs). Two of these RCTs were crossover studies. Due to significant heterogeneity, pooling the data into a meta-analysis was not possible.

Postprandial hyperinsulinaemic hypoglycaemia remains a notoriously difficult to manage metabolic complication of bariatric surgery. In this first systematic review, we present evidence suggesting that use of GLP-1RAs does not lead to an increase of hypoglycaemic episodes and although this approach may appear counterintuitive, our findings suggest that GLP-1RAs could reduce the number of postprandial hypoglycaemic episodes and improve glycaemic variability.

Disclosures: None

### P02 Increasing the number of referrals to the NHS Digital Weight Management Programme at a single GP centre - results from a two cycle audit

#### Sangha MS, Mehta N

##### University College London

The prevalence of people living with obesity is expected to reach 40% by 2035, the cost to the NHS is expected to reach ~£9.7billion by 2050. Primary care services can provide opportunities to help these patients. In the Herts CCG, NHS Digital Weight Management Service (NHS DWMS) offer free weight loss programmes for people with type 1/2 diabetes and/or hypertension with a body mass index (BMI) > 30, with each referral entitling the practice to Â£11.50. This study investigated the number of referrals made to NHS DWMS in a single GP centre and aimed to improve this using an electronic patient records pop-up and warning sign that would alert clinicians of patient eligibility for referral when reviewing electronic patient records prior, or during, consultations. From 27/12/21 to 20/02/22 (8 weeks) 325 eligible patients had GP appointments with only 19 (5.84%) being referred. After the electronic records pop-up was created, 63 of 354 patients (17.8%) were referred. This equates to the practice being entitled to an average of ~£90.56 a week (from ~£27.31 a week prior to pop-up intervention). It was also noted that referrals were more likely to be made for patients living with hypertension than patients living with diabetes (cycle 1: 7% and 2% respectively, cycle 2: 20% and 9% respectively). In our centre, a pop-up and warning system increases the number of eligible patients referred to NHS DWMS.

Disclosures: None

### P03 Anti-hypertrophic and Anti-inflammatory effects of Palmitoleic Acid in Adipose Tissue of Obese Mice with Repercussions in Liver

#### Simã JJ, Cruz MM, Armelin-Correa L, Alonso-Vale MIC

##### Federal University of Sã Paulo - UNIFESP, Brazil.

Our research has provided important evidence concerning the effects of palmitoleic acid, a natural omega-7 fatty acid (n7), which is abundant in plant, on metabolic disorders triggered by obesity. Considering the impact of obesity and related diseases on human health, studies focused on understanding the action of new agents that can modulate a healthy expansion of white adipose tissue (WAT) and treat related diseases are of relevance. Herein, we will show recent findings in WAT where n7 promoted metabolic changes and partially prevented in WAT the increase of gene expression that are triggered by obesity, suggesting that Obese+n7 animals do not require the same magnitude of metabolic adaptation to cope with energy demand from the high fat diet (HFD). Additionally, after 8 weeks of HFD-induced obesity in mice, we also observed that n7 had a beneficial effect on the changes trigged by HFD, attenuating the increase of lipids in liver, the body mass gain and reversing visceral adipocyte hypertrophy. However, n7 did not reverse the increase in WAT depot mass, suggesting greater hyperplasia in WAT of animals treated with n7. To further investigated, we used 3T3-L1 cell line, well characterized for adipogenesis studies. N7 increased proliferation, differentiation and the expression of Ppar-Î^3^2 and Cebpa, the master regulators of adipogenesis, as well as the expression of its target genes that encodes proteins necessary for the maintenance of the adipocyte phenotype. Finally, n7 decreased the expression of pro-inflammatory genes in adipose-derived stromal vascular fraction (SVF). Taken together, n7 modulates functional capabilities of SVF cells during the process of inflammation induced by HFD, and our data reinforces that there is a cross-talk between adipocytes and adipose-derived stromal cells to promoting n7 anti-hypertrophic and anti-inflammatory synergistic effects in obese mice. These results elucidate new agent and cellular targets to promote WAT healthy expansion.

Disclosures: None

### P04 Acceptance Commitment Therapy for Weight Loss: Insights from the clients- A Qualitative study

#### Ayva S, Edwards S

##### MoreLife UK

Rates of people living with obesity and overweight are rising in England (The King’sfund, 2021). It affects around one in every four adults (NHS, 2022). MoreLife UK delivers tailor-made, evidence-based, grounded in psychological and behaviour change theory health improvement programmes to individuals and families in area range of locations across the UK. We implemented a curriculum update in 2020 and expanded our intervention with elements of Acceptance and Commitment Therapy (ACT) tools and techniques.

ACT is an evidence-based third-wave therapy technique and has proven records of helping people with various mental health issues. The main aim of ACT is to help people live rich and meaningful lives (Hayes, 2019). ACT has been shown to facilitate longer-term behaviour changes compared with other techniques (Lillis, 2007; Heather et al., 2012; Fletcher, 2012; Reijonen, 2018; Fard et al., 2016; Forman et al., 2019).

This study aims to understand which ACT elements are perceived as helpful for weight management clients to lose weight and maintain their weight loss and how they facilitated their weight loss and management.

We will conduct two focus groups and four individual interviews with our clients to identify the unique contribution of ACT to the weight management programme. A focus group with the service practitioners to learn about their insights regarding the ACT elements of the programme and how these elements facilitate behaviour change and weight loss in their clients has already been undertaken. We will conduct a thematic analysis to identify patterns in the qualitative data.

We conducted a focus group with 6 of our practitioners. They believe that the values exercise, experiential avoidance, and urge surfing are helpful concepts of ACT. Results from the client feedback will follow.

Disclosures: None

### P05 Implementing a Guided Self-help Intervention for the management of Binge Eating prior to a Specialist Weight Management Programme

#### Edwards S, Gately P, Hill A, Traviss-Turner G

##### Leeds Beckett University

Binge eating is common in those living with obesity. NICE (2000) recommends Guided Self-Help (GSH) as a first line treatment for binge eating. However, services for binge eating are not widely available in the UK and many people experiencing binge eating are referred to weight management services. Binge eating poses a barrier to engagement with weight management and longer-term weight loss. The current service evaluation was a pilot to assess the preliminary effectiveness of delivering a brief, evidence-based GSH intervention for binge eating to adults, prior to them engaging in a standard NHS Tier 3 weight management service.

Service users were screened for binge eating using the Binge Eating Scale (BES). Those with a score ≥ 27 were invited to take part in the GSH intervention prior to engaging with the standard weight management programme. The intervention was based on cognitive behavioural principles. It comprised 7 sessions over 12 weeks and was supported by practitioners who had received appropriate training. 33 participants were found to be suitable, 22 started the programme, 9 dropped out and 13 have completed to date.

11 of the 13 participants who completed the GSH intervention reduced binge eating. Six had ceased binge eating altogether. The mean BES score pre-intervention was 34.3 (SD = 6.0) and post-intervention 20.6 (SD = 9.8). Of the 13 completers, 12 went on to engage in the standard weight management programme.

Commissioning psychological GSH support to address binge eating prior to engaging in Tier 3 weight management services shows promise. The intervention resulted in a reduction in mean BES scores representing a change from ‘severe binge eating’ to ‘mild or moderate binge eating’. This approach may reduce the pressure on eating disorder services and provide a more efficient pathway for those who wish to address both binge eating and complex obesity.

Disclosures: Paul Gately and Sophie Edwards are employed by MoreLife, the service commissioned to deliver the service

### P06 Outcomes at One Year in a Community Tier 3 Weight Management Service

#### Edwards S, Gately P, Costelloe E

##### Leeds Beckett University

Obesity continues to be a major issue in the UK. Tier 3 weight management services are commissioned by the NHS to support those with complex obesity and are often delivered in secondary care settings. Current literature indicates that Tier 3 services have a short- to mid- range improvement on weight and health for service users, but there is limited data on outcomes beyond six months. This service evaluation looks at the outcomes at one year for service users who have completed this community-based Tier 3 weight management programme.

Service users are referred by healthcare professionals and have a BMI > 35 with comorbidities or >40 with or without comorbidities. The multidisciplinary team include a psychologist, dietitian and exercise professional who assess and advice on those with complex needs. The programme is made up of 12 weekly, group-based sessions which include lifestyle education and psychological support. These sessions are followed by nine monthly sessions that seek to support ongoing behaviour change. Weight and psychological measures (PHQ-9 for depression symptoms and GAD-7 for anxiety symptoms) are collected at referral, and at 12 months.

Between April 2020 and March 2021, 55 clients completed the 12-month programme. Mean weight loss was 8.82kg (SD = 8.39) or 6.79% (SD = 6.27) of their original weight. 39 of the 55 (71%) lost over 3% of their original weight and (63%) of the 55 lost over 5% of their original weight. Mean reduction in PHQ-9 scores was 3.36 (SD = 4.18) and mean reduction in GAD-7 scores was 2.49 (SD = 4.41).

Tier 3 weight management services utilising a psychological, group-based approach can lead to significant improvements in both weight and mental health outcomes at one year. Services can be successfully delivered in community settings which may reduce costs associate with secondary care services and improve access for those who have challenges travelling.

Disclosures: All authors are employed by MoreLife, the provider commissioned to deliver this service

### P07 How is the NHS Low-Calorie Diet Programme expected to produce behavioural change to support diabetes remission: An examination of underpinning theory

#### Evans TSm Hawkes RE, Keyworth C, Newson L, Radley D, Hill A, Matu J, Ells LJ

##### Leeds Beckett University, University of Manchester, University of Leeds, Liverpool John Moores University

In 2020, the National Health Service Low-Calorie Diet Programme (NHS-LCD) was launched, piloting a Total Diet Replacement intervention with behaviour change support for people living with Type 2 Diabetes and excess weight. Four independent service providers were commissioned to design and deliver theoretically grounded programmes in localities across England.

To (1) develop a logic model detailing how the NHS-LCD programme is expected to produce changes in health behaviour, and (2) analyse and evaluate the use of behaviour change theory in providers’ NHS-LCD Programme designs.

A documentary review was conducted. Information was extracted from the NHS-LCD service specification documents on how the programme expected to produce outcomes. The Theory Coding Scheme was used to analyse theory use in providers’ programme designs documents.

The NHS-LCD logic model included techniques aimed at enhancing positive outcome expectations of programme participation and beliefs about social approval of behaviour change to facilitate programme uptake and behaviour change intentions. This was followed by techniques aimed at shaping knowledge and enhancing the ability of participants to self-regulate their health behaviours, alongside a supportive social environment and person-centred approach.

Application and type of behaviour change theory within providers’ programme designs varied: One provider explicitly linked theory to programme content; two providers linked 63% and 70% of intervention techniques to theory; and there was limited underpinning theory identified in the programme design documents for one of the providers.

The nature and extent of theory use underpinning the NHS-LCD varied greatly amongst service providers, with some but not all intervention techniques explicitly linked to theory. How this relates to outcomes across providers should be evaluated. It is recommended that explicit theory use in programme design and evidence of its implementation becomes a requirement of future NHS commissioning processes.

Disclosures: None

### P08 A qualitative study exploring participant experience of Obstetric Weight Management (OWM)

#### Shiplee GB

##### Leeds Beckett University

Excess weight in obstetric populations is associated with negative health outcomes for both mother and child. OWM is defined as weight management for women in varied stages of childbearing. Stages may include preconception, peri-natal or postnatal. To date, there is little consensus and limited research. This research project explores participant experience of OWM, with the wider goal of developing recommendations for improving OWM approaches and provision.

OWM is an increasing public health challenge in line with the global issue of increasing excess weight in populations. Excess weight in childbearing is highly associated with increased risk of unplanned pregnancy loss and prevalence of short- and long-term negative health outcomes for both mother and baby. With some social groups being at more risk than others. National and local efforts in the UK to support women reduce and manage excess weight to promote healthier pregnancies, have been considered ineffective. This is partly based on growing statistics of miscarriage and still birth and the prevalence of gestational diabetes. Previous research specifies participation in local services supporting OWM is poor. Suggesting accessibility and negative associations with health care professionals are factors in low engagement rates.

This ongoing research project adopts qualitative research methods. Six months of fieldwork was conducted, based on a unique combination of focus groups and individual interviews with both ‘real women and families’ and health professionals. While practitioner ethnography was used to capture the researcher’s, professional experiences based on observations of appointments with service users and reflections on professional meetings. An analysis of the data is underway highlighting preliminary findings. Contributing to a set of recommendations and considerations for services aiming to effectively support women and families with excess weight.

Disclosures: None

### P09 Re:Mission ‘An evaluation of the NHS Low Calorie Diet Programme’ early learning

#### Ells LJ, Homer C, Radley D, Drew K, Brown T, Marwood J, Logue J, Watson P, Jones S, Clare K

##### Leeds Beckett University

The NHS long term plan made a commitment to test a Low Calorie Diet (achieved via a total Diet Replacement programme) for people living with, or at risk of, obesity and type 2 diabetes. Ten pilot sites were initially recruited to test the NHS Low Calorie Diet programme, delivered using one of three different behaviour change support models: one to one, group or digital. As NHS England are collecting and analysing quantitative process and clinical impact data, an additional qualitative and economic evaluation was required.

To deliver a coproduced, comprehensive qualitative and economic evaluation of the NHS Low Calorie Diet pilot, that will be integrated with the NHSE quantitative analyses, to provide an enhanced understanding of the long-term cost-effectiveness of the programme and its implementation, equity, transferability and normalisation across broad and diverse populations.

Methods: A comprehensive mixed method evaluation, underpinned by a realist approach to determine what works, for whom, in what context, and why, delivered through a series of five interlinked work packages.

Early learning from the first year of the programme mobilisation and implementation will be presented, which will provide an overview of delivery context, qualitative findings from mobilisation staff, primary care and service provider interviews and focus groups, and emerging insights from a service user survey.

The evaluation findings are providing real time insights to inform ongoing service development and future procurement activity.

Disclosures: None

### P10 Supporting Weight Management during COVID-19 (SWiM-C): a randomised controlled trial of an ACT-based intervention

#### Mueller J, Richards R, Jones RA, Whittle F, Woolston J, Stubbings M, Sharp SJ, Griffin SJ, Bostock J, Hughes CA, Hill AJ, Ahern AL

##### University of Cambridge

We evaluated the effect of a web-based, acceptance-based guided self-help intervention which aims to prevent weight gain in adults with overweight or obesity during the COVID-19 pandemic (SWiM-C: Supporting Weight Management during COVID-19).

We randomised 388 participants (≥18 years, BMI ≥ 25kg/m2) to the SWiM-C intervention (n = 192) or a control group (n = 196). SWiM-C is based on acceptance and commitment therapy (ACT) and is delivered remotely via an online web platform (12 weekly modules) and contact via telephone and email with a trained, non-specialist coach. The control group received a leaflet on weight management and wellbeing during the pandemic. Participants completed online questionnaires at baseline, 4 months, and 12 months. The primary outcome was change in self-reported weight from baseline to 12 months; secondary outcomes were eating behaviour, experiential avoidance, mental health, wellbeing and physical activity.

At 12 months, the adjusted difference in weight between the SWiM-C group and the control group participants was -0.81kg (95% confidence interval [CI]: -2.24 to 0.61kg). SWiM-C participants reported a greater reduction in experiential avoidance (-2.45, 95% CI: -4.75 to -0.15), uncontrolled eating (-3.36, 95% CI: -5.66 to -1.06), and emotional eating (-4.14, 95% CI: -7.25 to -1.02), and an increase in physical activity (8.96, 95% CI: 0.29 to 17.62) compared to the control group. No differences in mental health or wellbeing were observed at 12 months.

Whilst the effect of the SWiM-C intervention on weight was inconclusive, SWiM-C improved eating behaviours, physical activity and psychological flexibility. These variables have been previously identified as determinants of successful weight management. Further refinement of the intervention is necessary to ensure meaningful effects on weight prior to implementation in practice.

Disclosures: FW, JW, MS, SJS and JB report no conflicts of interest. AJH has consulted for Slimming World. CAH reports payment or honoraria from Ethicon, NovoNordisk and International Medical Press for lectures, presentations, speakers bureaus, manuscript writing or educational events. JM and RR are Trustees for the Association of the Study of Obesity (unpaid roles). ALA and SJG are the chief investigators on two publicly funded (MRC, NIHR) trials where the intervention is provided by WW (formerly Weight Watchers) at no cost outside the submitted work.

### P11 The association between goal setting and weight loss outcomes: an observational analysis of real-world data

#### Wren G, Koutoukidis D, Scragg J, Whitman M, Jebb SA

##### University of Oxford

Goal setting may aid health-related behaviour change. The present study aimed to investigate the association between two measures of goals setting (percent weight loss goal and weight loss motivation) and weight change during a digital programme for weight loss, in a real-world setting.

This is a prospective longitudinal analysis of 36,794 UK adults with a BMI of â‰¥25 kg/m2, who participated in a 12-week digital behavioural programme for weight loss. The outcome was weight change at 24wks, assessed using mixed-model repeated-measures analyses to explore the effect of goal setting adjusted for known confounders. We also examined whether engagement acted as a mediator in the association between goals and weight loss.

Of the 36,794 participants who had weight readings at baseline, 13.1% (n = 4,818) reported weight at 24wks including a disproportionately high number of participants who were older and had lower baseline body mass index (p < 0.001). Most participants set goals of 5-10% of their initial body weight (64.3%), but weight loss at 24wks was greater for those who set goals of >10% compared to 5-10% (mean: 5.21kg; 95% CI: 5.01, 5.41; p < 0.001). There was no significant difference between goals of 5-10% and <5%. Appearance was most frequently reported as a motivational factor (40.1%) compared to health (27.6%), fitness (13.8%) or self-efficacy (18.5%), but at 24wks, health and fitness were associated with greater weight losses than appearance (mean: 1.40kg; 95% CI: 1.15, 1.65 and mean: 0.38kg; 95% CI: 0.05, 0.70, respectively). Engagement with programme components was a significant independent predictor of weight loss but not a mediator of the effect of goals on weight loss.

Setting larger goals and being motivated for health or fitness reasons were associated with greater weight loss. Randomised controlled trials of setting these types of goals are needed to confirm causality.

Disclosures: None

### P12 Effects of long-term consumption of n-6 PUFA rich diet on the rat hypothalamic proteome

#### Telles MM, Pedroso AP, Forcelini Machado MM, da Silva Júlio V, Oyama LM, Tashima AK, Ribeiro EB

##### Universidade Federal de Sao Paulo (UNIFESP)

The unbalanced consumption of polyunsaturated fatty acids (PUFAs) with high omega-6 (n-6)/omega-3 (n-3) ratio, characteristic of the Western diet, has been associated to inflammation and non-communicable chronic diseases. Indeed, n-6 PUFA has been reported to be more obesogenic and diabetogenic than other sources of fat or carbohydrates. Thus, the present study aimed to investigate the impact of long-term consumption of soybean oil-enriched hyperlipidic diet on the hypothalamic proteome of rats. 2-mo-old Wistar male rats were divided into 2 groups according to the 8-week diet protocol: HFD group, fed with the soybean oil-enriched high fat diet; Control group, fed with a standard chow diet (C). Food/caloric intake were evaluated weekly. Concluding the diet protocol, rats were euthanized, and hypothalami were removed and frozen at -80ÂºC until processing. Total protein content was extracted from hipothalami samples, and further analyzed by nanoLC-MS/MS. All the differentially expressed proteins were submitted to pathway analysis. Our results showed that the long-term consumption of soybean-enriched hyperlipidic diet modified hypothalamic proteins related to glucose metabolism, cytoskeleton remodeling, calcium signaling, Unfolded Proteins Response, endocytosis, exocytosis, and glutamate metabolism. Considering that most of the proteins altered were recognized to be involved in neurodegenerative processes, present findings suggest a potential harmful effect of the long-term consumption of soybean oil-enriched high fat diet. Therefore, further studies are needed to better comprehend the harmful potential of the overconsumption of n-6 PUFA on the pathogenesis of neurodegeneration.

Disclosures: None

### P13 Ginkgo biloba extract modulates hippocampal signaling pathways related to the regulation of feeding behavior in ovariectomized rats

#### Machado MMF, Banin RM, Thomaz FM, de Andrade IS, Hirata BKS, Ribeiro EB, Telles MM

##### Universidade Federal de Sao Paulo (UNIFESP)

Several studies have demonstrated that menopause contributes to the triggering of energy homeostasis disturbances, especially affecting feeding behavior, and thus favoring the development of obesity. However, even being observed improvement in these conditions after hormonal replacement therapy (HRT), alarming side effects are associated with this treatment, limiting its use by women with a history of breast cancer and cardiovascular disease. We have previously observed that Ginkgo biloba Extract (GbE) attenuated ovariectomy-related obesity, improving the hypophagic response of serotonin in the hypothalamus, and reducing serum leptin levels. Moreover, several anti-obesogenic properties of GbE were reported in diet-induced obese male rats, as well as its stimulatory effect on gene expression of hypothalamic anorexigenic effectors in normal male rats. Therefore, the present study investigated the effects GbE supplementation on hippocampal protein levels of 5-HT1A and 5-HT1B serotonin receptors, serotonin transporter (5-HTT), and leptin receptor (LepR) in ovariectomized rats. 2-month-old Wistar female rats were ovariectomized (OVX) or Sham-operated. After 2 months, daily oral gavages were performed once a day with 500 mg.kgâˆ’1 of GbE or vehicle for 14 days. GbE restored ovariectomy-induced decrease of 5-HT1A, and 5-HT1B protein levels in the hippocampus. Moreover, LepR hippocampal levels increased after GbE treatment, reaching similar levels of Sham rats. No changes were identified in 5-HTT levels. In summary, the present findings indicated that GbE improved the effectiveness of pivotal mechanisms involved in hippocampal generation of the negative feedback of food intake that were impaired by ovariectomy. Thus, GbE might be useful to alleviate disturbances related to energy homeostasis in menopause, which may also favor the improvement of body profile. Further studies are warranted to better understand the therapeutic potential of GbE in menopause.

Disclosures: None

### P14 A Systematic Review Of Physical Activity and Nutritional Interventions for The Management of Normal Weight and Overweight Obesity

#### Jacob E, Avery A

##### University of Nottingham

Normal Weight Obesity (NWO) is a highly prevalent, unclearly defined condition associated with increased cardiometabolic risk, and there are no systematic reviews conducted on its management. Overweight (BMI 25kg/m2 to < 30kg/m2) also has sparse research findings.

Systematically review physical activity and nutritional interventions for effectiveness and safety in the management of Normal Weight and Overweight Obesity (BMI < 30kg/m2 with a marker of raised body fat other than BMI).

Electronic databases were searched. Clinical trials including any physical activity or nutritional interventions, published between 2012-2022, evaluating body fat change were selected. Risk of bias was assessed.

Seven trials met inclusion criteria, including one single arm intervention, and six randomised controlled trials. A High Intensity Interval Training (HIIT) intervention with a high risk of bias had the largest effect on reducing body fat percentage (MD -5.18%, SD 0.14), with the next highest effects from high protein intake interventions (MD -3.70%, SD 1.47; MD -1.40%, SD 0.17). These three interventions also had the highest increase in lean mass. Two calorie restricted interventions had the highest mean weight loss (MD -3.10kg SD 0.87; MD -2.40kg, SD 0.25), but also had loss of lean mass/fat free mass, resulting in low reductions in body fat percentage (MD-1.10% SD 0.57; MD -1.26% SD 1.46). No serious adverse events were reported. There was considerable heterogeneity between studies.

There are physical activity and nutritional interventions that are effective for the management of normal weight and overweight obesity. There are numerous promising physical activity interventions. The most promising nutritional intervention is high protein intake and the least promising is energy restriction. More high quality, multi-intervention trials are urgently needed to assess effectiveness and safety of interventions, define target losses and the best tools to measure adiposity. Thanks to Rupesh Tailor for statistical assistance.

Disclosures: Dr Elizabeth Jacob is a Director of the company Dr Liya Jacob Ltd. Amanda Avery has a Consultant Role at Slimming World.

### P15 A critique of obesity strategies published by the Scottish Government since devolution

#### Steiner T, Craig L

##### University of Aberdeen

Overweight and obesity has remained a public health issue in Scotland for decades. Since devolution in 1998, Scottish Governments have released multiple strategies aimed at preventing obesity however rates have remained persistently high. It is fair to expect that a positive tangible impact would have been achieved by this point. This paper provides an evaluation of obesity strategies published in Scotland since devolution.

The study followed methods developed by Theis and White (2021) who previously carried out an evaluation of obesity strategies in England. Their comprehensive analytical framework in which policies within obesity strategies are coded by various themes was applied. Content analysis was completed by counting the frequencies of codes and the findings discussed using applied thematic analysis.

Scottish Governments have largely favoured low-intervention obesity policies. Policies generally lacked sufficient detail to explain how they would be implemented, and they also carried minimal levels of regulation to ensure delivery. Policies were found to be designed in a way that placed at least some level of responsibility on individuals in society. Finally, there was very little evidence of on-going learning and evaluation from strategy to strategy over time. However, the most recent obesity strategy did show signs of improved design.

Weak policy design and a lack of ongoing evaluation are likely to be key reasons behind the ineffectiveness of the strategies to reduce obesity rates. The Scottish Government must look to higher intervention policies, such as fiscal levers, if real improvement is to be achieved.

Disclosures: None

### P16 Primary Care Network Holistic Nutritionist and Health Coach - Remote and Supermarket Based Consultations, Impact on Lifestyle and Weight: A Service Evaluation

#### Haseler T, Nelson B, Maynard R, Mandicate K

##### Camden Central PCN

Primary care networks (PCNs) can bring innovative lifestyle and weight interventions to local populations with efficiencies of scale, particularly given developments in digital and remote working. Presently, evidence on feasibility or impact to support these services is lacking. Central Camden PCN developed a local pilot holistic health coaching intervention, delivered remotely and at local supermarkets, tailored towards targeting obesity, and functional symptoms. The aim of this poster is to evaluate this service’s feasibility and impact.

Patients were referred by primary care professionals (PCPs) to a British association for Nutrition and Lifestyle Medicine certified therapist. Inclusion criteria included patient goals of weight loss, symptom control, or ‘other’. Four one-to-one consultations over four months were offered, including education on food-labels and shopping for healthy food at the patient’s supermarket. Biometric data and patient self-assessment questionnaires were collected at the first and fourth consultations.

From February 2020 to April 2022 213 referrals were received, 33 patients completed the programme, 107 did not book their first appointment, the remainder have not completed their appointments. Of 33 completers, 6 were referred for a primary indication of weight loss, 13 for functional bowel symptoms, and 14 for ‘other’. 20 completers were female (60.6%), and 22 were aged 18-34 years, mean age 34.7. 15 patients had BMI > 25 on referral (mean Weight=90.7kg), with a mean decrease after four consultations of -2.3kg (2.3%, p = 0.005). Beneficial changes with p < 0.05 were also seen in fruit and vegetable intake, symptomatic scores and BMI. Wellbeing and activity levels did not alter significantly.

This PCN-level service is effective in engaging PCPs in referrals, and benefitting completers’ diets, weight and symptoms. There is, however, inequity of access, with a predominantly young and female completer population, and a low rate of referral-to-completion conversion which requires further evaluation.

Disclosures: None

### P17 Effectiveness of weight management interventions in adults delivered by primary care: A systematic review and meta-analysis of randomised controlled trials

#### Madigan CD, Graham HE, Sturgiss E, Kettle VE, Gokal K, Biddle G, Taylor GMJ, Daley AJ

##### Loughborough University

To examine the effectiveness of behavioural weight management interventions for adults delivered by primary care.

A systematic review and meta-analysis of randomised controlled trials.

Randomised controlled trials of behavioural weight management interventions delivered by primary care relative to comparator groups with weight change measured at >12-month follow-up. Participants were adults with a body mass index >25 kg/m2.

Trials from a previous systematic review were extracted and a search was completed using the following databases: Cochrane Central Register of Controlled Trials, MEDLINE, PubMed and PsychINFO from 1st January 2018 to 19th August 2021.

Data extraction and synthesis: Two reviewers independently identified eligible studies, extracted weight data, and assessed risk of bias using the Cochrane risk of bias tool. Meta-analyses were conducted with random effects models and a pooled mean difference for both weight (kg) and waist circumference (cm) were calculated.

Weight change from baseline to 12 months (primary), baseline to >24 months. Change in waist circumference was assessed at the same time points.

34 trials were included; 14 additional trials from the updated 2018 search. Twenty-seven trials (n = 8,000) were included in the primary outcome of weight change at 12-month follow-up. The mean difference between the intervention and comparator groups at 12 months was -2.3 kg (95% CI -3.0 to -1.6, I2 88%, p < 0.001), favouring the intervention group. At >24 months (13 trials, n = 5011) the mean difference was -1.8 kg (95% CI -2.8 to -0.8, I2 88%, p < 0.001) favouring the intervention. The mean difference in waist circumference was -2.5cm (18 trials, n = 5,288, 95% CI -3.2 to -1.8, I2 69%, P < 0.001) in favour of the intervention at 12 months.

Conclusions: Weight management interventions delivered in primary care are effective for weight loss and could be offered to help the public manage their weight.

Disclosures: None

### P18 The effect of maternal comsumption of green tea extract, by rats, during pregnancy and lactation, promotes lower evolution in body weight and regulates the cytokine replication in female offspring

#### Alves-Nakakura FC, Pontes LPP, Boldarine VT, Neto NIP, Feitoza AHS, Hachul ACL, Oller do Nascimento CM, Oyama LM

##### Universidade Federal de São Paulo

The type of maternal diet during the pregnancy period has an impacting role on the health evolution and risk of diseases in the offspring’s adult life. Green tea has anti-obesity, antioxidant, anti-inflammatory and immunomodulatory properties. In this context, the aim of the study was to investigate the effects of maternal consumption of green tea extract during pregnancy and lactation on the evolution in body weight and inflammatory profile markers in female offspring. Three-month-old female Wistar rats, during the 1st day of pregnancy up to the 28th day of lactation, were divided in two groups: MW = mothers that received water and ME = mothers who received green tea extract (400mg/kg of weight body weight/day), both with a growth control diet. After lactation, on day 28 postpartum, one female pup from each mother was euthanized and they were divided into the following groups: OW = offspring of the mother who received water and OE = offspring of the mother who received green tea extract. Pups body weight gain, adipose tissue depot weight and inflammatory markers protein in the adipose tissue were evaluated. When compared to OW animals, the OE group showed lower body weight gain and mesenteric adipose tissue relative weight. The MyD88 protein content was increased in the mesenteric adipose tissue. The parametrial adipose tissue presented an increase in the content of cytokines IL-6, TNF-Î ± and IL-1Î^2^. In summary, the results emphasize that the lower evolution in the body weight and the elevation of inflammatory profile makers in newborn can have harmful consequences for the health of the female offspring in adult life.

Disclosures: None

### P19 Effects of aerobic, resistance and concurrent training in the browning process in rats with obesity induced by hyperlipidic diet

#### Pontes LPP, Alves-Nakakura FC, Neto NIP, Boldarine VT, Maza PK, Avila F, Antunes HKM, Damaso AR, Oyama LM

##### Universidade Federal de São Paulo

Physical training has been widely used as a non-drug clinical strategy in the treatment of obesity. The objective was to evaluate the effect of resistance, aerobic and concurrent training on markers of browning of white adipose tissue from rats with obesity induced by a high fat diet. The experiment lasted 16 weeks, with eight weeks of obesity induction and eight weeks of physical training and change diet to a normocaloric one in male Wistar rats. The groups were Sedentary control diet (CS/n = 8), Sedentary high fat diet (HS/n = 8), Aerobic exercise (AE/n = 8), Resistance exercise (RE/n = 8) and concurrent training (CE/ n = 8). Body mass, food intake and analysis of browning process markers FGF-21, Irisin, PGC1Î ± , PPARÎ^3^ and UCP1 in subcutaneous (SUB), retroperitoneal (RET) and mesenteric (MES) adipose tissue were evaluated. The minimum level of significance adopted was 5%. The trained groups had lower adiposity and delta mass compared to the HS group. Irisin in RET adipose tissue was higher in the RE group compared to the HS group; in the MES, the AE group was higher in relation to the CS group; in SUB, the RE group was higher when compared to the CS group. The concentrations of FGF21 in the RET and SUB in the RE group were higher than in the control groups, respectively. The concentration of PGC1Î ± was higher in the RE group of adipose tissue from the MES and SUB when compared with the control groups; PPARÎ^3^ was higher in the RE group in the MES when compared to the HS group, and UCP1 showed higher levels in the AE in relation to the HS group. In conclusion, both resistance and aerobic training were efficient in activating different biomarkers of the browning process. However, no important adaptations resulting from concurrent training were observed.

Disclosures: None

### P20 Acceptance Commitment Therapy for Weight Loss: Insights from the clients- A Qualitative study

#### Sirin-Ayva, AB, Edwards, S

##### MoreLife UK

Rates of people living with obesity and overweight are rising in England (The King’s fund, 2021). It affects around one in every four adults (NHS, 2022). MoreLife UK delivers tailor-made, evidence-based which are grounded in psychological, and behaviour change theory health improvement programmes to individuals and families in area range of locations across the UK. We implemented a curriculum update in 2020 and expanded our intervention with elements of Acceptance and Commitment Therapy (ACT) tools and techniques.

ACT is an evidence-based third-wave therapy technique and has proven records of helping people with various mental health issues. The main aim of ACT is to help people to live rich and meaningful life (Hayes, 2019). ACT has been shown to facilitate longer-term behaviour changes compared with other techniques (Lillis, 2007; Heather et al., 2012; Fletcher, 2012; Reijonen, 2018; Fard et al., 2016; Forman et al., 2019).

This study aims to understand which elements of ACT are perceived as helpful for weight management clients to lose weight and maintain their weight loss and how these elements facilitated their weight loss and management.

We will conduct two focus groups and four individual interviews with our clients to identify the unique contribution of ACT to the weight management programme. A focus group with the service practitioners to learn about their insights regarding the ACT elements of the programme and how these elements facilitate behaviour change and weight loss in their clients has already been undertaken. We will conduct a thematic analysis to identify patterns in the qualitative data.

We conducted a focus group with 6 of our practitioners. They believe that the values of exercise, experiential avoidance, and urge surfing are helpful concepts of ACT. Results from the client feedback will follow.

Disclosures: None

### P21 Ginkgo biloba extract supplementation differently alters fatty acid composition in neutral lipid classes of the liver in obese rats fed a high-fat diet

#### Joyce EC ^1^, Hirata BKS ^2^, Machado MMF ^2^, Telles MM ^2^, Bueno AA ^1^

##### ^1^University of Worcester, ^2^Federal University of So Paulo Brazil

Obesity can result in metabolic disturbances, chronic inflammation and disturbed tissue fatty acid (FA) composition. The liver is dynamically involved in lipid metabolism including fatty acid β-oxidation and de novo lipogenesis. Antioxidant and anti-inflammatory properties have been attributed to Ginkgo biloba (GbE) supplementation. We investigated whether GbE supplementation altered neutral lipid FA profiles of liver tissue in high-fat-diet (HFD)-induced obese rats.

2-month-old male Wistar rats, were fed from 2 to 4-months-old with a HFD (28% lard), followed by 14 days of supplementation with saline (HFD-S) or GbE (HFD-GbE) at 500mg/kg. As GbE supplemented rats ingested fewer calories, a pairfed (HFD-PF) group was also included. Rats were euthanized and liver tissue removed. Total lipids were Folch-extracted and neutral lipids separated chromatographically into triglycerides (TAG), cholesteryl esters (CE) and monoglyceride + diglycerides (MAG + DAG), methylated and analysed by gas chromatography.

Liver TAG ω3 metabolite levels increased for HFD-PF (↑33%) and HFD-GbE (↑55%) compared to HFD-S, decreasing the ω6/ω3 ratio to 14:1 in HFD-GbE compared to HFD-S (19:1, p = 0.001) and HFD-PF (17:1, p = 0.02). CE-monounsaturate FA levels increased in HFD-GbE compared to HFD-S (p = 0.0001) and HFD-PF (p = 0.002) while HFD-GbE polyunsaturate FA levels decreased compared to HFD-S (p = 0.0003) and HFD-PF (p = 0.02). The CE ω6/ω3 ratio increased to 8:1 (p = 0.01) following GbE treatment compared to 5:1 in both HFD-S and HFD-PF. MAG + DAG-saturated FA levels decreased in HFD-PF (p = 0.01) and HFD-GbE (p = 0.003) compared to HFD-S, while MUFA levels increased in HFD-PF (p = 0.01) and HFD-GbE (p = 0.001).

The combined changes in liver TAG, CE and MAG + DAG FA levels, suggest GbE supplementation may contribute to altered liver lipid metabolism, peripheral FA store mobilisation or changes in ω3 and ω6 inflammation mitigation conferring some protection against the deleterious effects of a high fat diet. Further studies are needed for better understand of the beneficial effect of GbE.

Disclosures: None

### P22 A systematic review of the effect of digital game-based and influencer food and non-alcoholic beverage marketing on children and adolescents

#### Evans R, Maden M, Jones A, Christiansen P, Albadri S, Boyland E

##### University of Liverpool

Videogame livestreaming platforms are an emerging form of digital media where individuals can watch gaming influencers play videogames. These platforms are popular with children and targeted by food and non-alcoholic beverage (hereafter: food) brands, yet few studies have examined the impact of their food marketing exposure on children. Studies assessing the impact of television food marketing on children’s beliefs and behaviours map onto a logical hierarchy of effects linking food promotions to weight outcomes via brand awareness, attitudes and preferences, purchase, and consumption. This novel systematic review examined evidence for a relationship between exposure to food marketing within digital game-based media and via influencers, and these outcomes in young people.

Studies in which digital game-based or influencer food marketing exposure was experimentally manipulated, and at least one of the hierarchy of effects outcomes measured, in young people (up to 18 years) were included. Thirteen electronic databases were searched. Experimental (quantitative or mixed-method) and observational studies were considered. The review was pre-registered in PROSPERO [CRD42020167360] and conducted in accordance with PRISMA guidelines.

Twenty-two studies were included. Meta-analyses indicated an effect of food marketing on attitudes and preferences, and consumption behaviours. Purchase and awareness outcomes were synthesised narratively. Most included studies had either some concerns or a low risk of bias.

Evidence suggests that there is a relationship between exposure to food marketing via influencers and digital gaming media, and several hierarchy of effects outcomes. Findings are the first to demonstrate this relationship collectively, which has implications for food marketing policy.

Disclosures: P.C. has received research funding from the American Beverage Association. All such funding is for work outside of the current review.

### P23 Development and initial evaluation of a weight management programme tailored for people with serious mental illness: a non-randomised feasibility study with qualitative interviews

#### Lee C, Piernas C, Waite F, Aveyard A

##### University of Oxford

People with serious mental illness (SMI) have higher rates of obesity and premature mortality due to cardiovascular disease (CVD) than the general population. Trials show behavioural weight management programmes (BWMPs) can help people with SMI lose weight and reduce the burden of CVD. However diagnostic-specific barriers to uptake and engagement are reported. We aimed to develop and evaluate a standard BWMP tailored for people with SMI - called ‘Weight cHange for people with sErious mEntal iLlness (WHEEL).’

The development comprised: 1) 12 patient and public contributors with SMI; 2) a systematic review of qualitative studies to identify programme characteristics that promote uptake and engagement for SMI; 3) a systematic review of trials testing BWMPs to identify which characteristics lead to weight loss; and 4) coding the effective characteristics against a standard 12-week BWMP to identify opportunities for tailored support. Initial evaluation comprised: 5) a non-randomised study of feasibility (retention and n, % of programme sessions attended) and acceptability (qualitative interviews plus self-reported weight loss) at end-of-programme.

The programme developed was a weekly BWMP delivered by a commercial company. It was augmented with a one-off educational session geared towards people with SMI and weekly mentor check-ins. Seventeen participants (mean age: 48·52 years; 47% with schizophrenia) enrolled in the feasibility study and 16 were followed-up at 12-weeks (95% retention). All participants attended the educational session, 9/16 attended 50% of the weekly BWMP sessions, and 12/16 responded to 50% of the weekly check-ins. All participants reported weight loss (mean 4·06kg, SD: 3·17) and valued the novel education and therapeutic support. However anxious avoidance remained a barrier to joining the BWMP.

This study showed initial evidence that a standard BWMP augmented with brief education and low-intensity support is feasible, acceptable, and may lead to weight loss in people with SMI.

Disclosures: None

### P24 Sources of information and support preferred by people living with obesity (PLWO) in the ACTION-IO study UK

#### Hughes C, Ahern A, Halford JCG, McGowan B, Kasetty H, Vincent A, Parretti H

##### University of East Anglia, University of Cambridge, University of Leeds, Guys & St Thomas’ NHS Trust; Novo Nordisk UK

To explore the preferred sources of information and support for PLWO in the ACTION-IO study UK.

The ACTION-IO online survey was completed by PLWO (body mass index ≥30 kg/m2 based on self-reported height and weight) in 11 countries to investigate the perceptions, attitudes, behaviours and potential barriers to effective obesity care. We analysed data from the UK subset (N = 1500). Where appropriate, comparisons with global ACTION-IO data are reported.

81% of PLWO had discussed or would consider discussing weight with their healthcare professional (HCP). Only 21% who had discussed found it helpful, or very helpful; 69% acted on HCP advice, but only 39% thought they were somewhat successful; 26% of HCPs did not provide any suggestions. Improving diet and physical activity were the most frequent weight-management strategies. Few PLWO reported visiting an obesity specialist, using pharmacotherapy or bariatric surgery. The most frequent sources of information about managing weight were Internet (31%), family and friends (27%) and weight-loss programmes (26%). Only 23% cited HCPs (29% in the global study). The top three factors for improving weight loss were: HCPs providing weight-loss solutions directly to PLWO (70%), providing solutions to help HCPs treat PLWO (51%) and reducing misinformation about obesity (40%). About a third mentioned reducing stigma, changing HCP judgemental views and increasing recognition of obesity as a disease.

In the UK primary care, HCPs are the gateway to NHS weight-management services. PLWO were prepared to discuss weight with their HCP, but many did not find this helpful and wanted solutions delivered directly to patients. Treatment effectiveness might be improved by better education of HCPs, increased referral to specialist services, improved Internet- and media-based patient information resources, and offering PLWO direct access to effective weight-management interventions.

Disclosures: CH: Consultancy for Alva Health, Novo Nordisk; honoraria from Ethicon, Johnson & Johnson, Novo Nordisk and research grants from NIHR. AA: Scientific advisory board for Weight Watchers; Principal Investigator in two trials where Weight Watchers donated the intervention (no cost); research grants from the Medical Research Council and NIHR Programme Grants for Applied Research. JCGH: The University of Leeds has received consultancy income from Novo Nordisk and Dupont; all monies used to fund research. BMG: Consultancy and honoraria for Ipsen, Novo Nordisk; advisory for Lilly, Novo Nordisk; research grants from Novo Nordisk; shareholder Reset Health. HK: Novo Nordisk employee. AV: No conflicts of interest to declare. HP: Honoraria from Novo Nordisk and Johnson & Johnson; research grants from NIHR, UEA Health and Social Care Partners, PHE and OHID.

### P25 Does using planning policy to restrict new takeaways reduce childhood overweight, obesity and inequalities?: A quasi-experimental analysis of Gatesheadâ€™s Supplementary Planning Document between 2015-2020

#### Xiang H, Albani V, Goffe L, Nasima A, Lake AA, Wildman J, Brown H

##### Newcastle University

North-East England has a high prevalence of childhood obesity, with 29.1% of children in year 6 obese in 2020/21. Local authorities are responsible for improving population health but have limited budgets and capacity. Gateshead introduced supplementary planning guidelines in 2015 that banned any new takeaways in the local authority. Our previous research found that this policy led to a reduction in the density and proportion of fast-food outlets. However, its impact on health was unknown.

In this study, we investigate changes in the density and proportion of takeaways by area deprivation and if this has led to decreases in inequalities in childhood overweight and obesity prevalence.

We used Middle Super Output Layer (MSOA) data on childhood overweight and obesity from the National Child Measurement Programme from 2011-2020. We used food outlet data from the Food Standard Agency Food Hygiene Rating Scheme for 2012-2020 and data on Index of Multiple Deprivation 2015 from Office of National Statistics. We employ a quasi-experimental method, a difference-in-difference approach, to compare changes in childhood overweight and obesity rates between Gateshead and five other local authorities in the North-East of England which did not have planning guidance restricting new takeaways.

We only found a significant reduction of 2.7% (95% CI: -5.3% to -0.2%) in year 6 overweight in the second most deprived quartile of MSOAs in Gateshead compared with local authorities which did not adopt any planning policy. This may be because of heterogeneity in the number of takeaways in each decile of deprivation.

Limiting new takeaways through planning policy may have helped contribute to reducing health inequalities in childhood weight in Gateshead. As planning policy is dynamic it is important to consider how this evidence may help other local authorities tackle obesity.

Disclosures: None

### P26 Spillover effects from early childhood, dietary obesity prevention strategies: A rapid review

#### Marr C, Breeze P, Caton S

##### University of Sheffield

Interventions targeting early childhood obesity are often-family based, as such they have the potential to not only impact upon the target participants but also on other family members. Changes in outcomes for those not directly targeted in an intervention can be referred to as spillover effects. The aim of this rapid review was to explore the spillover effects from early childhood, dietary-based obesity prevention strategies. Medline, Web of Science and Psycinfo were searched in July 2020. 12 studies met the inclusion criteria and were included in the review. In most of the studies (n = 11) the intervention targeted multiple health behaviours incorporating dietary components. Spillover effects were only captured for parents of children enrolled in an obesity prevention intervention. Dietary outcomes were the most frequently explored spillover effect, followed by weight based outcomes. There were significant positive effects of early childhood obesity prevention strategies on parental fruit and vegetable intake. Results were mixed for spillover effects on parental sugar intake and dietary pattern scores. Of the five studies capturing parental weight outcomes only one study found significant positive changes in parental weight. Although spillover effects often accompanied significant changes to the weight or diet of the child targeted by the intervention, in three studies, spillover effects occurred irrespective of significant positive changes in child outcomes. Obesity prevention strategies targeting children in the early years can also benefit other members of the family. Failure to capture spillover effects from early childhood obesity prevention strategies may underestimate intervention effectiveness and cost-effectiveness. Further work is needed to explore the spillover effects from early childhood obesity prevention strategies on other family members and caregivers, such as siblings and grandparents.

Disclosures: None

### P27 Illustrative cases of approach to and management of GI complication post bariatric surgery

#### Zarkasi ZA, Hazlehurst JM

##### Department of Diabetes and Endocrinology, University Hospitals Birmingham NHS Foundation Trust, Birmingham, UK

Interventions targeting early childhood obesity are often-family based, as such they have the potential to not only impact upon the target participants but also on other family members. Changes in outcomes for those not directly targeted in an intervention can be referred to as spillover effects. The aim of this rapid review was to explore the spillover effects from early childhood, dietary-based obesity prevention strategies. Medline, Web of Science and Psycinfo were searched in July 2020. 12 studies met the inclusion criteria and were included in the review. In most of the studies (n = 11) the intervention targeted multiple health behaviours incorporating dietary components. Spillover effects were only captured for parents of children enrolled in an obesity prevention intervention. Dietary outcomes were the most frequently explored spillover effect, followed by weight based outcomes. There were significant positive effects of early childhood obesity prevention strategies on parental fruit and vegetable intake. Results were mixed for spillover effects on parental sugar intake and dietary pattern scores. Of the five studies capturing parental weight outcomes only one study found significant positive changes in parental weight. Although spillover effects often accompanied significant changes to the weight or diet of the child targeted by the intervention, in three studies, spillover effects occurred irrespective of significant positive changes in child outcomes. Obesity prevention strategies targeting children in the early years can also benefit other members of the family. Failure to capture spillover effects from early childhood obesity prevention strategies may underestimate intervention effectiveness and cost-effectiveness. Further work is needed to explore the spillover effects from early childhood obesity prevention strategies on other family members and caregivers, such as siblings and grandparents.

Disclosures: None

### P28 The effect of maternal comsumption of green tea extract, by rats, during pregnancy and lactation, promotes lower evolution in body weight and regulates the cytokine replication in female offspring

#### Alves-Nakakura FC, Pontes LPP, Boldarine VT, Neto NIP, Feitoza AHS, Telles MM, Hachul ACL, Oller do Nascimento CM, Oyama LM

##### Universidade Federal de São Paulo

The type of maternal diet during the pregnancy period has an impacting role on the health evolution and risk of diseases in the offspring’s adult life. Green tea has anti-obesity, antioxidant, anti-inflammatory and immunomodulatory properties. In this context, the aim of the study was to investigate the effects of maternal consumption of green tea extract during pregnancy and lactation on the evolution in body weight and inflammatory profile markers in female offspring. Three-month-old female Wistar rats, during the 1st day of pregnancy up to the 28th day of lactation, were divided in two groups: MW = mothers that received water and ME = mothers who received green tea extract (400mg/kg of body weight/day), both with a growth control diet. After lactation, on day 28 postpartum, one female pup from each mother was euthanized and they were divided into the following groups: OW = offspring of the mother who received water and OE = offspring of the mother who received green tea extract. Pups body weight gain, adipose tissue depot weight and inflammatory markers protein in the adipose tissue were evaluated. When compared to OW animals, the OE group showed lower body weight gain and mesenteric adipose tissue relative weight. The MyD88 protein content was increased in the mesenteric adipose tissue. The parametrial adipose tissue presented an increase in the content of cytokines IL-6, TNF-Î ± and IL-1Î^2^. In summary, the results demonstrate a metabolic programming aspect of green tea extract ingestion during pregnancy and lactation which could promote metabolic disarrangement in an adulthood life.

Disclosures: None

### P29 Perceived barriers and facilitators to healthy school food provision and the young personal food choice: A mixed methods study

#### Rose K, Lake A, O’Malley C, Brown L, Ells L

##### Teesside University

The reported eating behaviours and nutrition intake of adolescents in the UK call for urgent policy attention. Irregular eating patterns and inadequate consumption of low fruit and vegetables, indicate that young people are not achieving the required nutrients for growth, development, and immune purposes. Schools are identified as a supportive ‘place’ setting where nutrition outcomes of young people can be shaped and influenced. Moreover, the COVID-19 pandemic has exposed and potentially worsened issues to enable health promotion in the school environment. This research aimed to explore the views and perspectives of those involved in school food to further understand the influences on the young person’s (11-18 years) food choices, including consideration of COVID-19 specific school guidance.

A mixed method study was undertaken. Interviews were carried out with school leadership and surveys to catering staff in four schools in the North East of England and a national survey distributed to young people, parents, carers and staff in the UK. Responses were analysed using a Thematic analysis approach. Themes represented barriers and facilitators to healthy school food promotion and provision which may influence food choice.

Results revealed there are multiple barriers to healthy school food promotion and provision in secondary schools in the UK. Findings suggest that context matters, and schools in more deprived areas are against the tide of poor nutrition, behaviour and low aspirations from early years. Poor access to healthy foods and easy access to HFSS impact eating habits. COVID-19 has exasperated issues faced when attempting to improve the nutritional intake of young people with guidelines highlighting challenges schools meet in their attempts to serve healthy food. Additional support is needed for schools to successfully implement school food standards, incorporate nutrition education and behaviour change techniques. Evaluation of school food standards and adherence in UK schools is recommended.

Disclosures: None

### P30 A Practical toolkit for schools: What good looks like; A whole school food approach

#### Rose K, Lake A,O’Malley C, Lalli G

##### Teesside University

Dietary intake and reported eating behaviours of adolescents in the UK are of increasing public health concern. Schools are identified as an ideal setting to promote health and improve young peoples’ nutrition outcomes. A gap in the understanding of how healthy secondary school food policy can be implemented, sustainably and effectively, may hamper progress to improving school food provision and nutrition education in the UK. Research was conducted to explore factors which influence healthy school food provision and adolescent food choice. Findings were utilised to develop a practical toolkit to enable schools to implement a whole school food approach.

The research incorporated an exploration of the secondary school food environment as a potentially ‘obesogenic’ setting, the effectiveness of school food interventions and policy in Europe and UK. Priority was given to young people’s (11-18 years) eating behaviours and preferences in food choice. A pragmatic approach was taken with the integration of evidence, incorporating a mixed method approach. This demonstrated influences on young people’s eating behaviours at multiple levels, including data supporting the effectiveness of European school food interventions and policies.

A 6-phase practical toolkit is presented, guided by ‘What Good Looks Like’ and ‘Whole Systems Approach to Obesity’ principles which can be used to translate the evidence into practice in schools. Improving secondary school food provision across the school day and having a coherent whole school approach to healthy eating has the potential to significantly improve a young person’s food choice, therefore impacting on the nutrient intake of adolescents in the UK.

Disclosures: None

### P31 Psychological support within behaviour changing (Tier 2) Weight Management Services: are we doing enough?

#### Marwood J, Brown TJ, Kaiseler M, Clare K, Feeley A, Blackshaw J, Ells LJ

##### Leeds Beckett University

A significant proportion of people live with obesity and mental health issues such as disordered eating, or mild forms of anxiety and depression. There is an urgent need to understand the experiences and priorities of people living with obesity and mental health issues to ensure more person-centred obesity care.

To identify what psychological support is provided in adult Tier 2 behavioural Weight Management Services (T2 WMS) by asking weight management service users, commissioners, and providers their views on the use of, and need for, psychological support.

An online (Qualtrics) survey of users, providers and commissioners of T2 WMS was conducted. Service users were recruited through the Obesity UK support group, advertisement on Twitter and via service providers. Commissioners and providers were recruited through the Office for Health Improvement and Disparities (OHID) and local authority networks. Quantitative data was summarised and described, and open-ended questions were coded, and themes drawn out.

Participants were individuals who were current or recent (last 5 years) users of T2 WMS (n = 27), commissioners (n = 9) and providers (n = 17).

Service users reported eating to cope with stress or emotions, and that poor mental health impacted motivation to change. Service users described a bidirectional relationship between weight and mental health and the difficulties of navigating complex healthcare systems. Over half of service users did not feel their mental health needs were met, and 60% said they would like additional psychological support within T2 WMS. However, there was a lack of consensus over what psychological support entailed, and it was often confused with behaviour change techniques.

This survey highlights service user perception of the links between mental health and ability to manage weight, and the need for appropriate, person-centred screening, triage and psychological support within T2 WMS.

Disclosures: None

### P32 Changes in health-related quality of life and depressive symptoms in the first year following bariatric surgery: the BARI-LIFESTYLE observational study

#### Jassil FC, Carnemolla A, Kingett H, Doyle J, Lewis N, Montagut-Pino G, Kirk A, Marvasti P, Chaiyasoot K, Zakeri R, Mok J, Brown A, Elkalaawy M, Jenkinson A, Adamo M, Devalia K, Parmar C, Batterham RL

##### University College London

The desire to improve quality of life is one of the factors that motivates people to seek bariatric surgery. Currently, there is a paucity of prospective UK studies assessing the impact of bariatric surgery on health-related quality of life (HRQoL) and depressive symptoms.

Patients undergoing bariatric surgery at three NHS trusts were enrolled in the BARI-LIFESTYLE observational study and received post-bariatric standard care. HRQoL and depressive symptoms were assessed using EuroQol-5Dimensions-3Levels (EQ-5D-3L), Impact of Weight on Quality of Life-Lite (IWQoL-Lite) and Beck Depression Inventory-II (BDI-II) questionnaires at pre- and post-surgery (3-, 6- and 12-month). Anthropometric and objective physical activity data were also collected.

Prospective data from 77 patients (80.5% female) with a mean ± SD age of 43.4 ± 10.6 years and body mass index of 42.9 ± 5.8 kg/m^2^ were analysed. The EQ-5D-Index and EQ-Visual Analogue Score improved significantly post-surgery, mean (95% CI) improvement of 0.01 index (0.01 to 0.02) and 2.1% (1.7 to 2.5), both p < 0.001, respectively. Similarly, the post-surgery total IWQoL-Lite score increased significantly, 3.4% (2.9 to 3.8), p < 0.001, relative to the pre-surgery score. The BDI-II scores also showed significant improvement over time, -0.8 points (-0.9 to -0.6), p < 0.001. The improvement in HRQoL and depressive symptoms peaked in the first three months post-surgery. In multivariate linear regression, factors such as percentage weight loss (%WL), type of bariatric procedure and time spent on moderate-to-vigorous physical activity (MVPA) were found to mediate the improvement in HRQoL. At six months post-surgery, participants with no or minimal to mild depressive symptoms had significantly higher %WL compared to participants with moderate to severe depressive symptoms (21.3 ± 5.2 versus 15.6 ± 2.7%, p = 0.001).

Bariatric surgery improves HRQoL and depressive symptoms. The link between time spent on MVPA and HRQoL requires further investigation. Future studies should also elucidate the relationship between post-surgery depressive symptoms and weight loss outcomes.

Disclosures: None

### P33 The Contribution of a Dietitian to a Midwifery-led Healthy Pregnancy Clinic

#### Adams O, Cutter J

##### Cardiff and Vale UHB

This is a presentation of service development in early years as part of the Healthy Weight: Healthy Wales strategy, 2019.

The Welsh Government produced the ‘Health Weight: Healthy Wales strategy’ in 2019 and they then identified seven national priority areas in their strategy ‘Healthy Weight: Healthy Wales: Moving Ahead in 2022-2024’. Priority three within this strategy is to promote and support families to provide the best start in life, from pre-pregnancy to early years. The need for early intervention and prevention was identified as an important part of the strategy ensuring that, where appropriate, expectant parents across Wales will have access to services to help manage their weight.

Cardiff and Vale University Health Board deliver a Midwifery led Healthy Pregnancy clinic which supports pregnant women with a BMI between 35 and 39.9 kg/m^2^, and no co-morbidities, the choice to deliver on a Midwifery led unit.

A Dietitian joined the Healthy Pregnancy Clinic in November 2021 following the identification of this priority within the weight management strategy. The Dietitian delivers an intervention for any woman with a BMI > 40 kg/m^2^, identified at their booking appointment. They are offered an initial 30-minute consultation within the antenatal clinic and this is a chance to share their thoughts about their weight, to discuss weight gain in pregnancy and to look at options for weight management beyond pregnancy. The Dietitian also discusses entering the Adult weight management service pathway, post-partum. The focus is on engagement initially and uses the skills and intention of Motivational Interviewing throughout the consultations.

In addition to working in collaboration with the Midwives in the Healthy Pregnancy Clinic, the Dietitian delivers training sessions on healthy eating in pregnancy, raising the topic of weight with pregnant women and using Motivational Interviewing skills for helpful conversations on lifestyle change.

Disclosures: Orla Adams receives payment for work with RCGP and Novo Nordisk

### P34 An Evaluation of the Nutrition and BMI Clinical Link Pathway in Mental Health and Learning Disabilities Services

#### Stevens H, Giles EL, Smith J, Bussey L, Lake AA,McGeechan GJ

##### Teesside University

The aim of this research was to evaluate the Nutrition and Body Mass Index Clinical Link Pathway (NBMI CLiP) in practice, which is one element of the A Weight Off Your Mind intervention. AWoYM focuses on maintenance of a healthy lifestyle and weight management and is delivered to service users living with severe mental illness and/or learning disabilities across Tees, Esk and Wear Valleys NHS Foundation Trust (TEWV).

The evaluation utilised a mixed-methods approach. Quantitative analysis of routine data was used to assess change in three key performance indicators and opinions were collected from staff implementing the pathway via an online survey and one-to-one interviews. Additional data was collected on staffs’ own weight management.

Secondary data analysis showed the majority of individual wards had improved recording of the three key performance indicators. Qualitative findings from the survey and interviews indicated that the majority of participants who used the NBMI CLiP thought it easy to use. They were unaware that TEWV offered staff weight management groups but felt that they would be interested in attending one. Comments were made regarding the lack of healthy food provision for inpatients and staff across TEWV. It was also felt that there was a lack of accessible materials available for patients.

It was recommended that training on the NBMI CLiP should be delivered during induction for new members of staff, with regular refresher sessions. A revision of food provision for staff and inpatients across TEWV should focus on the availability of healthier options and staff training on nutrition. The NBMI CLiP materials require assessment and revision to reflect accessibility standards.

Disclosures: None

### P35 Semaglutide 2.4 mg Reduces the 10-Year Risk of Type 2 Diabetes in People with Overweight/Obesity

#### Hazelhurst J, Garvey WT, Holst-hansen T, Laursen PN, Rinnov AR, Wilkinson LJ

##### University Hospital Birmingham, University of Alabama at Birmingham USA, Novo Nordisk A/S, Denmark; Novo Nordisk, USA

The effect of once-weekly subcutaneous (s.c.) semaglutide 2.4 mg on the risk of developing type 2 diabetes (T2D) in people with obesity is unknown.

Weight management with semaglutide versus placebo plus diet/exercise was assessed in participants with overweight/obesity in STEP 1 (68 weeks) and STEP 4 (20-week run-in with all participants treated with semaglutide, 48-week randomised withdrawal, during which participants either continued semaglutide or switched to placebo). The 10-year T2D risk was calculated post hoc using Cardiometabolic Disease Staging (CMDS), a validated Bayesian logistic regression of T2D risk factors (age, sex, race, body mass index, triglycerides, high-density lipoprotein, blood pressure and fasting plasma glucose).

Risk scores decreased after 68 weeks from 18% to 7% with semaglutide, and from 18% to 16% with placebo (61% vs 13% reduction [p < 0.01]; STEP 1). Most of the risk score reduction with semaglutide occurred during weeks 0-20, from 21% to 11%; risk score decreased further to 8% with continued semaglutide during weeks 20-68, but increased to 15% with switch to placebo (32% reduction vs 41% increase [p < 0.01]; STEP 4). Week 0 risk scores were higher in participants with prediabetes versus normoglycaemia, but treatment effects were comparable at week 68 (p = 0.45 for interaction [STEP 1]). Risk score changes mirrored weight loss, which was 17% with semaglutide versus 3% with placebo in STEP 1, and in STEP 4 was 11% for weeks 0-20 with semaglutide, and a further 9% with continued semaglutide versus a 6% regain with switch to placebo for weeks 20-68.

Treatment with semaglutide reduces the 10-year risk of T2D by ~60% regardless of initial glycaemic status, with sustained treatment required to maintain this benefit. These data suggest that semaglutide could help prevent T2D in people with obesity.

Disclosures: JH: honoraria from Novo Nordisk and funding from NIHR RfPB and NIHR CRN. WTG: relationships with Boehringer Ingelheim International GmbH, Eli Lilly and Company, Epitomee, JAZZ Pharmaceuticals, Novo Nordisk, Pfizer Inc. TH-H: Novo Nordisk A/S employee and shareholder. PNL: Novo Nordisk A/S employee. ARR and LJW: Novo Nordisk employees.

### P36 A qualitative exploration of barriers and facilitators of weight loss during participation in an online behavioural weight loss programme

#### Thomson M, Martin A, Long E, Logue J, Simpson SA

##### University of Glasgow

Behavioural weight loss programmes are efficacious in improving health and weight outcomes in adults living with obesity. Despite their success, many participants still struggle to achieve their weight loss goals. Exploring the influence of environmental, interpersonal, and intrapersonal factors during participation in programmes can highlight barriers and facilitators to success. This study aimed to compare successful and unsuccessful participants in the factors reported as influencing weight loss.

Semi-structured interviews were conducted with 48 participants. Participants were predominantly female (83%) with a mean age of 49.09 (+/-10.16) years and a mean BMI of 31.60 (+/-4.80). Interviews included questions on how their environment, social life, and internal factors had affected their weight loss journey. Interviews were analysed in NVivo using a thematic approach. Following coding and completion of the programme, participants were grouped as successful (weight loss of >5%) or unsuccessful and the emergent themes were compared between groups.

The interviews revealed that successful and unsuccessful participants shared many barriers and facilitators. Barriers included: accessibility to resources, poor social dynamics, negative thoughts, and feelings and habitual behaviours with food and alcohol. Facilitators included positive social influence and support, embedding behavioural change into daily life, improvements in wellbeing, and positive physiological response. When comparing differences between the groups, unsuccessful participants were less pragmatic in finding solutions to barriers, less aware of their weight and health, and experienced more negative social experiences (e.g., conflict or less support).

These findings provide novel insights into the factors participants themselves identify as hindering or facilitating their weight loss, as well as key differences between those who were successful and unsuccessful. Future programmes and research should consider these findings and incorporate strategies to increase success rates

Disclosures: None

### P37 Women’s experiences of a moderately reduced-carbohydrate intervention designed to help prevent gestational diabetes: a descriptive qualitative study

#### Michalopoulou M, Jebb SA, Astbury NM

##### University of Oxford

Reducing carbohydrate intake can help control glucose levels and weight gain in women with obesity who are pregnant and at risk of gestational diabetes mellitus (GDM). However, womenâ€™s experience of following such a programme from early or mid-pregnancy is unclear. We therefore interviewed participants from the REduced-Carbohydrate intervention for the management of Obesity and Reduction of gestational Diabetes (RECORD) feasibility trial.

We conducted semi-structured in-depth telephone interviews with a purposive sample of sixteen participants who were randomised to the RECORD trial intervention group. Audio-recordings were transcribed verbatim. Questions focused on participantsâ€™ experience of the RECORD diet and of the study. We conducted a descriptive thematic analysis using the one sheet of paper (OSOP) method.

Eight themes were identified; Reasons for taking part, Understanding GDM, Acceptability, Barriers and Facilitators, Adherence and Sustainability, Support and Accountability, Motivation, Outcomes. The health of the fetus as an incentive to make healthier dietary choices was central to many of the themes. About half of the participants had not thought about GDM before the study. Participantsâ€™ first perceptions of the dietary changes varied from doubt or worry, through to acceptance and enthusiasm. Engagement with the diet was facilitated by clear messages, structured resources, and use of behavioural strategies. Regular follow-up by the counsellor and objective measures helped sustain dietary changes. Support from social environment and the counsellor, as well as personal accountability were important in increasing motivation. In a few cases however, pregnancy-related physical and psychological problems, negative body image, and history of dieting, impeded adherence and motivation. Participants reported similar behavioural changes and positive physical and psychosocial impact. All participants were content with their taking part in the study.

The majority of women at risk of GDM reported that a reduced-carbohydrate programme delivered alongside antenatal appointments was overall a positive experience.

Disclosures: None

### P38 Trends In Weight Loss Attempts Among Children in England

#### Ahmed A, Little M, Piernas C, Jebb SA

##### University of Oxford

There has been a steady rise in the prevalence of overweight and obesity in English children over the last decade and despite childhood obesity being named as a government priority in 2004, there is little knowledge of the prevalence or demographic characteristics of children attempting to lose weight. The objective of this paper is to describe trends in reported weight loss attempts among school-aged children in England and to investigate its sociodemographic determinants.

We analysed data of children aged 8-17 years who participated in the Health Survey for England (HSE) from 1997-2016 (n = 34,235). This repeated cross-sectional survey reported weight loss attempts and sociodemographic characteristics. Body weight and height were measured by trained interviewers and BMI for age z-score was calculated. Multivariable logistic regression was used to investigate the sociodemographic determinants. The main outcome measures were weight loss attempts by year, age group, gender, weight status, ethnicity, and household income.

The prevalence of reported weight loss attempts increased significantly from 21.4% (1997-98) to 26.5% (2015-16). The increase was significant for boys, older children, Asian children, children from lower income households and in all categories of BMI for age z-score. Significant predictors of weight loss attempts included having overweight (8-12 years old, OR:4.01 [%CI:3.47, 4.64]; 13-17 years old, OR:1.96 [%CI:1.58, 2.42]) or obesity (8-12 years old, OR:13.57 [%CI:11.94, 15.43]; 13-17 years old, OR:4.72 [%CI:3.94, 5.66]) as well as being older girls, from ethnic minority groups or low household income.

The prevalence of reported weight loss attempts among children is increasing at a faster rate than the rise in excess weight and includes an increasing proportion of children with a ‘healthy’ weight. The increase in the prevalence of reported weight loss attempts among children is greatest among subgroups with lower baseline prevalence.

Disclosures: None

### P39 The effectiveness of interventions to promote a healthy weight around pregnancy: an overview of systematic reviews

#### Matu J, Griffiths A, Shannon O, Brown T, Jones A, Swann C, Davison M, Ells L

##### Leeds Beckett University

Excessive gestational weight gain has been associated with foetal and maternal complications, with higher risks in those living with greater degrees of excess weight. Identifying feasible, acceptable, and effective strategies which can help women achieve a healthy weight around the time of pregnancy, and encourage appropriate gestational weight gain, is therefore desirable and could help to reduce the risk of adverse outcomes during and after pregnancy.

The Cochrane Library, MEDLINE, PsycINFO and CINAHL were searched from 1st January 2000 to 29th July 2021. Articles eligible for inclusion were published systematic reviews which included randomised or nonrandomised controlled intervention studies investigating the effectiveness of interventions to promote a healthy weight around pregnancy (up to 3 months prepregnancy, during pregnancy, or up to 12 months postpartum) with an assessment of maternal weight status before and after the intervention.

A total of 2773 records were identified, 11 of which were included in this overview. Gestational weight gain was significantly lower in the intervention group compared with the control group in all identified reviews which assessed diet only (n = 4). Four out of the five included reviews which assessed physical activity only interventions showed a reduced GWG compared with control. Four out of six reviews which assessed combined diet and physical activity interventions showed a significantly reduced gestational weight gain compared with control. One review found a significantly lower gestational weight gain with behavioural and counselling interventions compared with control in mothers living with obesity. Of the 11 included reviews, seven were classified as “critically low”, two were classified as “low”, and two were classified as “high”, using the AMSTAR 2 critical appraisal tool.

Both single component, and multi-component interventions have potential to reduce gestational weight gain in individuals around the time of pregnancy.

Disclosures: None

### P40 The feasibility and acceptability of evaluating the Change4Life Food Scanner app in reducing children’s sugar intake

#### Mahdi S, Chilcott J, Buckland NJ

##### University of Sheffield

The Change4Life Food Scanner app provides nutritional information on packaged foods using images such as sugar cubes. The app’s effectiveness for improving dietary choices is unknown. This study investigated the feasibility and acceptability of evaluating the effectiveness of the Food Scanner app in reducing children’s sugar intake. 126 parents of 4-11 year olds were randomised into a Food Scanner app exposure condition (n = 62), or no intervention control (n = 64). Participants completed baseline and 3-month post-intervention measures of dietary intake using myfood24 and both open- and closed-ended trial acceptability measures. The intervention arm additionally completed fortnightly app engagement measures and provided app feedback. 64 participants (51%) completed the study (intervention: n = 29; control: n = 35). Of those, 80% reported that the study was easy to complete and 97% found task completion reminders helpful. However, 27% reported that food diaries were too much work and open-ended responses suggested limitations in myfood24. App engagement (minutes) decreased throughout the study (week 2: M = 18.01, SD = 27.15; week 12: M = 6.76; SD = 11.56). 86% reported high acceptability for the app’s use of sugar cube images, which were rated as easy to understand and useful to supplement front of package nutritional labels. However, 71% had low acceptability of the app for aiding food purchasing decisions. App improvement suggestions included a scanning progress chart, healthier substitute recommendations, and access to discounts for healthier alternatives. Additionally, 24% reported that COVID-19 impacted trial engagement. Open-ended responses suggested this was due to time constraints, and changes in usual dietary habits. 73% were willing to continue with the study for a 12-month trial. No significant differences in study acceptability ratings were found between control and intervention. Alongside suggested improvements to the Food Scanner app, findings from this research, in addition to ongoing analysis of preliminary efficacy of the intervention, will inform design parameters for a future large-scaled trial.

Disclosures: None

### P41 Impacts of Covid-19 lockdown on children’s BMI, physical fitness and health-related quality of life eighteen months after restrictions eased: a longitudinal study

#### Basterfield L, Galna B, Burn N, Weston KL

##### Newcastle University

The lockdowns implemented in 2020 and 2021 in response to the COVID-19 pandemic reduced free movement of populations. For children, school closures and disruption to their daily routine may have been detrimental to their physical and mental wellbeing. This study aimed to assess children’s body mass index (BMI), physical fitness and health-related quality of life (HRQoL) in 2019, 2020 and 2021.

In n = 79 children (mean 8.6 years at baseline), age- and sex-adjusted BMI (z-score), cardiorespiratory (20m shuttle run test: 20mSRT) and muscular fitness (standing broad jump: SBJ, handgrip strength: HGS, flexibility: sit-and-reach) and HRQoL (Kidscreen-27) were measured in October 2019 (baseline), November 2020 (T1) and November 2021 (T2). Longitudinal associations were assessed with repeat-measures ANOVA with post hoc t-tests.

Mean BMI z-score increased from 0.67 at baseline to 0.91 at T1 (p < 0.001) with no change at T2 (0.88, p = 0.518). Larger baseline BMI z-score predicted greater increase at follow-up. 20mSRT decreased from 23.1 shuttles at baseline to 20.4 at T1 (p = 0.034) with no change at T2 (24.4, p = 0.187). Sit-and-reach decreased from baseline to T1 (16.2cm to 14.1cm p < 0.001) to 16.0cm at T2 (p = 0.591). SBJ increased from 118cm at baseline to 127cm (p < 0.001) at T1 to 130cm (p < 0.001) at T2. Right HGS increased from 12.8kg at baseline to 14.1kg at T1 (p < 0.001) to 16.3kg (p < 0.001) at T2. A greater reduction in shuttles and SBJ predicted a greater increase in BMI z-score. HRQoL results were mixed, but ‘Physical Wellbeing’ decreased at each timepoint, ‘Parents and Autonomy’ increased from T1 to T2.

In this population of children, BMI z-score and cardiorespiratory fitness were adversely affected after the first lockdown. One year later, although some improvements were seen, they were not in line with the results expected during non-pandemic times. Future pandemic planning must acknowledge the long-term detrimental impacts of lockdowns on children’s health.

Disclosures: None

### P42 Two-year Effect of Semaglutide 2.4 mg vs Placebo in Adults with Overweight or Obesity: STEP-5

#### Papamargaritis D, Garvey WT, Batterham RL, Bhatta M, Buscemi S, Christensen LN, Frias JP, Jódar E, Kandler K, Rigas G, Wadden TA, Wharton S

##### University of Leicester, University of Alabama at Birmingham USA, University College London, UCLH

The 2-year efficacy and safety of once-weekly (OW) subcutaneous semaglutide 2.4mg versus placebo in adults with overweight/obesity was assessed in the STEP-5 phase-3 trial (NCT03693430).

STEP-5 was a randomised, double-blind, placebo-controlled trial in 5 countries. Adults with body mass index (BMI) ≥ 30kg/m2, or ≥27kg/m2 with ≥1 weight-related comorbidity, without diabetes, were randomised 1:1 to 104 weeks’ treatment with semaglutide 2.4mg or placebo. The co-primary endpoints were percent change in body weight (BW) and achievement of weight loss ≥5%. Cardiometabolic risk factors and safety/tolerability were also assessed. Data shown are for the treatment policy estimand (effects regardless of treatment adherence and use of other anti-obesity therapies). P-values for parameters marked with # were not controlled for multiplicity.

Results: In total, 304 adults were randomised (78% female; 93% white; mean age 47 years, BW 106.0kg, and BMI 38.5kg/m2). Mean BW change from baseline to week 104 was −15.2% with semaglutide vs −2.6% with placebo (estimated treatment difference: −12.6 %-points; 95% confidence interval: −15.3, −9.8; p < 0.0001). Participants were more likely to lose ≥5%, ≥10%, ≥15%, and ≥20%# BW with semaglutide vs placebo (77.1% vs 34.4%, 61.8% vs 13.3%, 52.1% vs 7.0%, and 36.1% vs 2.3%, respectively; p < 0.0001 for all odds ratios). Greater improvements were seen with semaglutide versus placebo in waist circumference, BMI#, systolic and diastolic# blood pressure, HbA1c#, fasting plasma glucose#, fasting serum insulin#, C-reactive protein#, and lipids# (total cholesterol, very-low-density lipoprotein cholesterol, and triglycerides) (p < 0.05 for all). No new safety signals with semaglutide were observed.

Subcutaneous semaglutide 2.4mg OW for 2 years resulted in substantial and sustained BW reductions and improvements in cardiometabolic risk factors versus placebo, indicating a favourable benefit-risk profile of semaglutide 2.4mg for long-term management of weight and cardiometabolic risk factors.

Disclosures: DP: grants from Novo Nordisk UK, Novo Nordisk A/S, Health Education East Midlands and Academy of Medical Sciences. WT: Relationships with Boehringer Ingelheim International GmbH, Eli Lilly and Company, Epitomee, JAZZ Pharmaceuticals, Novo Nordisk, Pfizer Inc. RLB: Research grant support from Novo Nordisk, consultancy for Boehringer Ingelheim, Eli Lilly, Glia Therapeutics Ltd, GSK, Novo Nordisk and Pfizer. MB, LNC and KK: Novo Nordisk A/S employees. SB: Principal Investigator for clinical trial NCT03693430 (no financial compensation, nor financial relationship). Advisory/consulting fees and/or other support from Boehringer Ingelheim, Eli Lilly, Guidotti Laboratories, Menarini Diagnostics, Novo Nordisk and Therascience Lignaform. JPF: Research support grants from Novo Nordisk, grants and personal fees from Boehringer Ingelheim, Eli Lilly, Merck, Novo Nordisk and Sanofi, and grants from Janssen and Pfizer. EJ: Grants from Amgen, AstraZeneca, Boehringer Ingelheim, FAES, Janssen, Lilly, MSD, Novo Nordisk, Pfizer, Sanofi, Shire and UCB, personal fees from Amgen, AstraZeneca, FAES, Helios-Fresenius, Italfúrmaco, Lilly, MSD, Novo Nordisk, UCB and Viatris. GR: advisory/consultancy and lecture fees and non-financial support from iNova Pharmaceuticals, Nestle HealthScience, Johnson & Johnson and Novo Nordisk, lecture fees from Medtronic (formerly Covidien), ReShape LifeSciences (formerly Apollo-Endosurgery and Allergen Australia), W.L. Gore Device Technologies and Merk Sharpe and Dohme. TAW: Advisory boards for Novo Nordisk and WW (formerly Weight Watchers) and grant support, on behalf of the University of Pennsylvania, from Novo Nordisk. SW: Research funding, advisory/consulting fees and/or other support from AstraZeneca, Bausch Health Inc., Boehringer Ingelheim, CIHR, Janssen, Lilly and Novo Nordisk.

### P43 Continuous Glucose Monitoring to assess the Glycaemic Variation in children and young people with Obesity

#### Apperley LJ, Parkinson J, Senniappan S

##### Alder Hey Children’s Hospital, Liverpool.

Childhood obesity is a known risk factor for developing type 2 diabetes mellitus. At present, the standard modality for diagnosis is an oral glucose tolerance test (OGTT). Continuous glucose monitoring (CGM) is used predominantly by patients with type 1 diabetes mellitus to monitor their glucose levels.

The aim of the study was to investigate glycaemic variation using CGM in children and young people with obesity, who have had no evidence of pre-diabetes or type 2 diabetes on OGTT.

Children and young people (aged 2-18 years) with obesity (BMI SDS > 2) who have had a recent normal OGTT were recruited. Free-living blinded CGM was commenced for a minimum of three days using Dexcom G6 devices.

In total, 13 patients were studied with a mean age of 14.4years (range:10.3-16.6). The average BMI was 40.2kg/m2 (+7.3SD) and mean BMI SDS was +3.5 (+0.5SD). The average HbA1c was 34mmol/mol (5.3%). The CGM devices were worn for an average of 8.0 days (range:3.4-11.9). The mean average glucose of all patients was 6.3mmol/L (+1.2SD) and average coefficient of variation was 19.8% (range: 14.3-45.9; normal <36%). Percentage time in and out of range showed a median time between 3.9 and 7.8 mmol/L (70-140mg/dL) of 83.5% (IQR 79.9-93.7). The median time with glucose levels >7.8 mmol/L (140mg/dL) and >10.0 mmol/L (180mg/dL) was 12.5% (IQR 2.5-16.7) and 0.1% (IQR 0-1.6), respectively.

The results show that the median time spent in target glucose range was 83.5%, which is below the expected 95% seen in healthy, non-diabetic participants, and that patientsâ€™ glucose levels are going above 10mmol/L (180mg/dL) at times. Our data has shown evidence of glycaemic dysregulation in children and young people with obesity who have had a normal OGTT. The results show a potential role for CGM in identifying glycaemic impairments earlier than conventional investigations.

Disclosures: None

### P44 Semaglutide 2.4 mg Induces Weight Loss and Improves Body Composition Across Age Groups in Adults With Overweight or Obesity: Post-Hoc Analysis of the STEP 1 Trial

#### Stuart K, Dicker D, Batterham RL, Frias JP, van Gaal LF, Jensen C, Lau DC, Laursen PN, McGowan BM, Kandler K, Greenway FL

##### Leeds Teaching Hospitals, Hasharon Hospital-Rabin Medical Center, Tel Aviv University, University College London

Body weight loss and body composition changes may differ by age. This post-hoc analysis of STEP 1 evaluated the effect of subcutaneous semaglutide 2.4mg on weight loss and body composition according to age.

Adults without type 2 diabetes with body mass index (BMI) ≥ 27kg/m^2^, with ≥1 weight-related comorbidity, or ≥30kg/m^2^ were randomised to once-weekly semaglutide 2.4mg or placebo, plus lifestyle intervention, for 68 weeks. Body weight change (%) was analysed according to age ≤40 (n = 655), >40-<60 (n = 981) and ≥60 years (n = 325). Proportions of body fat mass and lean body mass were assessed in a subgroup of 140 adults with BMI ≤ 40kg/m2. Body composition (% body fat mass, % lean body mass, lean:fat mass ratio) was analysed according to age <50 and ≥50 years.

Results: Body weight change (%) was greater for semaglutide versus placebo, consistent across age groups ≤40, >40-<60 and ≥60 years (estimated treatment difference [ETD] [95%CI]: − 13.0 [−14.6, −11.4], −12.1 [−13.4, −10.8] and −12.3 [−14.4, −10.1], respectively), with no interaction between treatment and age (p = 0.718). Semaglutide was associated with reduced % body fat mass and increased % lean body mass in people aged <50 (ETD [95%CI]: − 4.3 [−6.9, −1.6] and 3.9 [1.4, 6.3]) and ≥50 years (ETD [95%CI]: − 2.4 [−4.5, −0.3] and 2.1 [0.1, 4.0]) vs placebo. The ETD (semaglutide versus placebo) for change in lean:fat mass ratio at week 68 was 0.29 (95%CI 0.10, 0.47) for age <50 years, and 0.14 (95%CI -0.01, 0.29) for age ≥50 years, with no interaction between treatment and age for body composition change.

Conclusion: In STEP 1, weight loss and reduced % body fat mass/increased % lean body mass with once-weekly semaglutide 2.4mg versus placebo was achieved, with no treatment difference across age groups.

Disclosures: KS: Honoraria from Novo Nordisk.DD: research funding from AstraZeneca, Boehringer Ingelheim, Eli Lilly, Novo Nordisk and Sanofi-Aventis, consultant, advisory board member and speaker for AstraZeneca, BoehringerIngelheim, Eli Lilly, Merck Sharp & Dohme, Novo Nordisk and Sanofi-Aventis. RB: consulting fees from Boehringer Ingelheim, GSK, Novo Nordisk and Pfizer; research investigator for Novo Nordisk, speaker for ViiV and Novo Nordisk. JPF: Research support grants from Novo Nordisk, grants and personal fees from Boehringer Ingelheim, Eli Lilly, Merck, Novo Nordisk and Sanofi, and grants from Janssen and Pfizer. LvG: advisory board and/or speaker’s bureau of AstraZeneca, Boehringer Ingelheim,Lilly, MSD and Novo Nordisk. CJ: Novo Nordisk employee. DL: consultant for Amgen, AstraZeneca, Bausch Health, Boehringer-Ingelheim, Eli Lilly, HLSTherapeutics and Novo Nordisk. PL: Novo Nordisk A/S employee. BMG: advisory boards for Boehringer Ingelheim and Novo Nordisk, grants for educational work from Biogen, Boehringer Ingelheim, J&J, Janssen, Lilly, Novo Nordisk, MSD, Orexigen,Sanofi, and institutional research grant support from Novo Nordisk. Shareholder in Reset Health. KK: Novo Nordisk A/S employee. FLG: research funding from Melior Discoveries and NovMetaPharma; consultant for Basic Research and General Nutrition Corporation; advisory board for Nutraceutical Corp and Altimmune; stock/stock options in Ketogenic Health Systems Inc., Plensat, Rejuvenate Bio and UR Laboratories.

### P45 A model for treating obesity as a multimorbid condition: finding the â€œbest fitâ€: a systematic review protocol

#### Li R, Logue J, Li X

##### Lancaster University

With the high prevalence of comorbidities, obesity needs to be treated as a multimorbid chronic condition. However, there is no agreed model for managing obesity or multimorbidity. The aim of this systematic review is to identify the “best fit” models for the management of obesity-related multimorbidity.

Six databases will be systematically searched to identify existing care models in the management of multimorbidity. The eligibility of studies will be assessed by two reviewers. The definition of multimorbidity used will be a patient living with two or more conditions, where at least one condition is physical. The model will be defined as comprehensive interventions for managing multimorbidity simultaneously and lifelong. The interventions will have at least two components and have a clear theoretical basis. The components summarized from the identified model will be used to form a priori framework themes by using the ‘best fit’ framework synthesis (BFFS), and a new model will be created for the management of obesity-related multimorbidity

Models which have a high quality evidence base and evidence of effectiveness identified by this review identified a “best fit”model for the management of chronic multiple conditions, which will be taken forward to future work on potential models of care for obesity-related multimorbidity.

Disclosures: None

### P46 Exploring the time-course of cerebral blood flow changes after acute exercise in healthy men using functional neuroimaging: implications for food cue reactivity paradigms

#### Dera AM, Thackray AE, Alanazi TM, Hinton EC, King JA, Morgan P, Stensel DJ

##### Loughborough University, Jeddah University, King Saud bin Abdulaziz University for Health Sciences, University of Bristol, University of Nottingham, Waseda University Japan

Acute exercise has been shown to alter food cue reactivity using blood-oxygen-level-dependent (BOLD) functional magnetic resonance imaging (fMRI). Changes in cerebral blood flow (CBF) immediately after exercise may influence the BOLD signal during appetite-related paradigms. This analysis explored the time-course of CBF changes after exercise to identify the optimum time for food cue-related BOLD acquisitions.

Twenty-three men (mean(SD): 24(4) years, 22.9(2.1) kg·m-2) completed an exercise (60-mins running, 68(3)% peak oxygen uptake) and control (60-mins rest) trial in a randomised crossover design. Appetite ratings were assessed at five pre-identified time-points. Five-minute pseudo-continuous arterial spin labelling MRI scans were performed for CBF assessment before and at four consecutive repeat acquisitions after exercise/rest. Differences in CBF in grey matter and seven regions shown previously to respond to food cues were examined with 2Ã—5 (trial-by-time) repeated-measures ANOVAs using a non-parametric permutation approach (Randomise, FSL).

A main effect of trial identified greater CBF in the exercise than control trial in grey matter and the amygdala, hippocampus, insula and lateral orbitofrontal cortex (OFC) (all p ≤ 0.014). Regional CBF in the medial OFC and striatum was lower in the exercise than control trial (both p ≤ 0.003). Trial-by-time interactions were not identified in grey matter or the seven regions of interest (all p ≥ 0.087). The exercise-induced change in time-averaged area under the curve for fullness was positively correlated with the change in striatum and hippocampus CBF (r ≥ 0.517, p ≤ 0.012).

While overall between-trial differences in grey matter and regional CBF were evident, the time-course of CBF was not influenced directly by exercise suggesting food cue-related BOLD acquisitions in the immediate post-exercise period may not be time-sensitive. Future studies should consider acquiring concurrent BOLD and CBF data to account for between-trial differences in CBF that could confound the interpretation of brain food cue reactivity.

Disclosures: None

### P47 What we Learned from A Specific Healthy Lifestyle Pilot for Individuals living with Learning Disabilities

#### Edwards S, Godfrey J, Payne M, Edrich R

##### MoreLife UK

Individuals living with learning disabilities are at increased risk of being overweight or living with obesity compared to the general population (gov.uk, 2020). On the other hand, achieving healthier diets and healthier weights is one of the top priorities for Public Health England (PHE Strategy 2020 to 2025). There is a need to tailor weight management interventions for adults living with learning disabilities and associated conditions (Lally, et al. 2022).

The main purpose of this pilot study is to develop a specific healthy lifestyle programme for individuals living with learning disabilities. This is a pilot programme aimed to produce a tailored programme that fits the specific needs of individuals living with learning disabilities. This pilot is funded by Suffolk County Council and implemented by OneLife Suffolk.

To obtain insights from individuals living with learning disabilities, we conducted two focus groups: one with ACE-Anglia Independent Advocacy Organisation and the second one with Papworth Trust. With the help of these focus groups’ insights and relevant research publications, we created a flexible programme to deliver to individuals living with learning disabilities. The programme consists of 12 weekly sessions, and the activity levels are varied to meet mild to moderate learning disability levels. We delivered two Face to Face and one online group as well as twelve 121 interventions.

The programme is still rolling. We have 19 starters and 11 completers so far. All of the completers are in the monthly maintenance phase. Ten participants out of 19 are female. We collected both qualitative and quantitative data from the participants to have a clear understanding of their experience of the programme.

Average weight loss is -2.59% ranging between -10.40% to -0.1%. In addition to weight loss, the participants also mentioned the positives of learning about healthy eating, physical activity and nutrition.

Disclosures: None

### P48 A Novel Response to Delivering Peer Group Support During The Covid-19 pandemic

#### Clare K, Fullan J, Burbridge B

##### Obesity UK

With the sudden onset of the Covid-19 pandemic Obesity UK was forced to close its face to face support offering across the UK. In the early days advice was unclear though many members did receive to shield. It was clear that people living with obesity had increased risk of contracting the virus, had poor outcomes and increased mortality.

We swiftly established a twice weekly support group. One session focusses on the bariatric surgery pathway and the other focusses around more general support. In order to help sustain the input of volunteers we have introduced frequent speaker sessions these have included people with lived experience of obesity sharing a personal account of their lives and journey thus far.

We have been fortunate to engage academics and clinicians to come along on a Wednesday and share their expertise.

We will present some of our findings and lessons learnt. We can see a clear application for this model particularly outside centres of population where groups have traditionally been difficult to establish due to distances involved.

We wish to share the experiences and learnings from our speaker sessions we have made recordings available within our community in a media showcase and plan to extend this to a wider audience with our launch at UKCO.

Disclosures: KC has recieved personal fees from Novo Nordisk Boehringer Ingelheim and Apollo Endosurgery

### P49 Participants experiences of an online weight management service during the COVID-19 pandemic

#### Gill J, Ferrier K, Shearer R, Huang J, Zhang X

##### University of Glasgow, NHS Scotland

The COVID pandemic necessitated a transition of the Tier 3 weight management service delivered by the Specialist Weight Management Service (SWMS) in Glasgow from face-to-face to online delivery. We sought to investigate patients’ experiences of online delivery to guide service improvement. 177 patients attending the SWMS were sent an email link to an anonymous questionnaire comprising 36 closed and 4 open-ended questions, covering sociodemographic and background variables, reasons for wanting to lose weight, confidence in using technology, experiences of the online programme, comparisons with the face-to-face programme, and suggestions for service improvement. 68 patients, with a mean age of 53 years, responded. 71% were obese before the age of 30 years and 44% had been trying to lose weight > 20 years. Most commonly cited reasons for weight loss were to improve health (91%), improve mobility (88%), and improve quality of life (78%). Less than 20% reported that they struggled to engage in the programme due to limited technological skills or poor internet access. 58% of participants felt the online sessions provided the support that they needed for weight management and 61% would recommend the programme to friends or family. However, >50% found it more difficult to engage with discussions online, particularly for sensitive issues, compared to face-to-face and over two-thirds missed the opportunity for face-to-face interactions with healthcare professionals and their fellow participants. In terms of improvements, 64% would prefer access to tutorial education videos ahead of the session, and 46% would prefer the sessions to be more interactive. Going forward, 62% expressed preference for blended online and face-to-face delivery, with 29% preferring a fully face-to-face service. Thus, the online delivery appeared to be generally well-received by patients, and with some enhancements, could play a long-term role in weight management delivery as part of a blended service.

Disclosures: None

### P50 Patients with Type 2 Diabetes Reach Glycaemic Targets Faster with Tirzepatide Compared to Semaglutide and Titrated Insulin Degludec

#### Lynch A ^1^, Pantalone KM ^2^, Viljoen A ^3^,Galindo RJ ^4^, Cui X ^5^, Huh R ^5^, Fernández L ^5^, Patel H ^5^

##### ^1^Eli Lilly and Company, UK, ^2^Cleveland Clinic, USA, ^3^North & East Hertfordshire Hospitals Trust, ^4^Emory University School of Medicine USA, ^5^Eli Lilly and Company USA

Tirzepatide (TZP), a novel GIP/GLP-1 receptor agonist approved in the US for treatment of type 2 diabetes (T2D). TZP 5mg, 10mg, and 15mg demonstrated superiority versus semaglutide 1mg (SEMA) and titrated insulin degludec (iDeg) in HbA1c change from baseline and proportion of patients reaching HbA1c < 53mmol/mol (7%) and≤48mmol/mol (6.5%) at 40-weeks (SURPASS-2) and 52-weeks (SURPASS-3), respectively.

In SURPASS-2 and 3, TZP was initiated at 2.5mg once weekly and increased by 2.5mg every 4 weeks until the assigned dose was reached and maintained for the duration of the study. SEMA was initiated at 0.25mg once weekly and the dose doubled every 4 weeks until 1mg was reached and maintained for the duration of the study. iDeg was initiated at 10U/day and titrated weekly to a fasting blood glucose of <5.0mmol/L following a treat-to-target algorithm

In an exploratory pre-planned analysis, time to achieve glycaemic targets was estimated using Kaplan Meier method and hazard ratio between treatment was calculated using cox proportional-hazards model.

TZP was faster than SEMA and iDeg in time to reach HbA1c < 53mmol/mol (7%) and ≤48mmol/mol (6.5%). Median time to achieve HbA1c < 53mmol/mol (7%) was 8.1 weeks for all TZP doses versus 12.0 weeks for SEMA, and to reach ≤48mmol/mol (6.5%) was 12.1 weeks versus 15.7 weeks, respectively. TZP was faster than SEMA in time to lose 5% body weight. Consistently, median time to reach HbA1c < 53mmol/mol (7%) was 8.1 weeks for all TZP doses versus 12.1 weeks for iDeg, and to reach ≤48mmol/mol (6.5%) was 12.1 weeks versus 24.1 weeks, respectively.

Mild to moderate gastrointestinal adverse events were associated with TZP and primarily occurred during the dose escalation period and decreased over time.

In conclusion, patients with T2D reached glycaemic targets faster with TZP compared to SEMA 1 mg and titrated iDeg.

Disclosures**:** KMP reports grants from Twinhealth; personal fees from AstraZeneca, Sanofi, Corcept Therapeutics, Eli Lilly and Company, and Diasome; grants and personal fees from Bayer, Novo Nordisk, and Merck & Co. AV reports grants from Sanofi, consulting fees from Eli Lilly and Company, Novartis, Novo Nordisk, and Amarin; payment or honoraria from Boehringer, Eli Lilly and Company, Novartis, Napp, Novo Nordisk, Sanofi, Daiichi Sankyo, and Astra Zeneca; payment for expert testimony from Daiichi Sankyo; and travel support from Daiichi Sankyo, Napp, and Novo Nordisk. RJG reports grants from Eli Lilly and Company, Novo Nordisk, and Dexcom; consulting fees from Eli Lilly and Company, Weight Watchers, Sanofi, and BI; and travel support from Eli Lilly and Company. XC, RH, LFL, and HP are employees and shareholders of Eli Lilly and Company. AL (Non-author Presenter) is an employee and shareholder of Eli Lilly and Company.

### P51 Associations between maternal adiposity measures in early pregnancy and adverse maternal and infant outcomes: a systematic review and meta-analysis

#### Nguyen G^1^, Hayes L^1^, Ngongalah L^1^, Bigirumurame T^1^, Gaudet L^2^, Odeniyi A^1^, Flynn A^3^, Crowe L^1^, Skidmore R^4^, Simon A^5^, Smith V^6^, Heslehurst N^1^

##### ^1^Newcastle University, ^2^Queen’s University, Canada, ^3^Kings College London, ^4^University of Ottawa, Canada, ^6^Northumbria University

Maternal obesity increases the risks of adverse pregnancy outcomes. BMI > 30kg/m2 is routinely used to identify women requiring high-risk antenatal care. BMI is poor at predicting individual risk compared with alternative measurements (e.g. waist circumference). We aimed to explore associations between maternal adiposity and adverse pregnancy outcomes.

This systematic review was registered on PROSPERO (CRD42017064464). Searches included six databases (MEDLINE, EMBASE, PsycINFO, CINAHL (EBSCO), JBI Database of Systematic Reviews and Implementation Reports and Cochrane Library), references, citations and contacting authors. Screening, data extraction and quality assessment using the Newcastle-Ottawa Scale were carried out by two authors independently. Random-effects meta-analysis was performed when data were suitable for pooling, otherwise, narrative synthesis was conducted.

24,027 studies were identified following the removal of duplicates, 90 studies (n = 115,940 pregnancies) met the inclusion criteria and were included, reporting both maternal and infant outcomes. There were significantly increased odds of gestational diabetes (GDM) with higher waist circumference (WC) categories (1.40, 95%CI 1.04-1.88) and per unit increase in WC (1.31, 95%CI 1.03-1.67). Women with GDM had higher WC than controls (MD 6.18cm, 95%CI 3.92-8.44). WC was significantly associated with hypertensive disorders, delivery-related outcomes, metabolic syndrome, composite pregnancy outcomes and macrosomia. Maternal fat-free-mass (FFM) was associated with increasing birthweight (AE 18.07g, 95%CI 12.75-23.38). Waist-to-hip (WHR) ratio was significantly associated with GDM, hypertensive disorders and delivery-related outcomes. Fat mass, neck circumference, skinfolds, and visceral fat were significantly associated with adverse outcomes, although limited data were available.

Some measures of maternal adiposity may be useful for risk prediction of adverse pregnancy outcomes: WC, WHR, FFM. Further research is needed to directly compare adiposity measures and BMI performance at individual risk prediction to inform clinical practice.

Disclosures: None

### P52 Long-term Efficacy of Setmelanotide in Patients With POMC or LEPR Deficiency Obesity

#### Clément K ^1,2^, Wabitsch M ^3^, Van den Akker E ^4^, Argente J ^5,6^, Scimia C ^7^, Srinivasan M ^7^, Yuan G ^7^, Kühnen P ^8^

##### ^1 2^Inserm, NutriOmics Research Unit France, ^3^University of Ulm Germany, ^4^Erasmus University Medical Center Netherlands, ^5^Universidad Autónoma de Madrid, ^7^Rhythm Pharmaceuticals USA, ^8^Pediatric Endo, Berlin Germany

In Phase 3 trials, treatment with the melanocortin-4 receptor (MC4R) agonist setmelanotide led to weight reduction in participants with obesity due to biallelic variants in the genes encoding proopiomelanocortin (POMC), proprotein convertase subtilisin/kexin type 1 (PCSK1), or leptin receptor (LEPR). The current analysis assesses the long-term efficacy of setmelanotide in this population over ~3 years.

Patients with POMC, PCSK1, and LEPR biallelic deficiency aged ≥6 years who demonstrated clinical benefit and acceptable safety after treatment with setmelanotide (up to ~12 months) in a prior (index) trial were eligible to continue treatment in this long-term extension (LTE) trial (NCT03651765). Body weight measures and safety/tolerability were assessed for up to 3 years. The current analysis reports outcomes relative to index trial baseline.

Of 28 patients enrolled in the index trial, 21 and 15 received ≥24 and ≥36 months of treatment in the LTE, respectively. At index trial baseline, mean (standard deviation [SD]) body mass index (BMI) for all patients was 44.9 (11.8) kg/m2, body weight in patients aged ≥18 years was 147.7 (28.7) kg, and BMI Z score in patients age <18 years was 3.6 (0.4). After 24 and 36 months, mean (SD) percent change in BMI was −16.7% (16.0%) and −17.5% (20.5%), respectively. Mean (SD) percent change in body weight in those aged ≥18 years was −16.7% (16.2%; n = 10) and −13.5% (15.9%; n = 8) after 24 and 36 months of treatment, respectively. Mean (SD) change in BMI Z score in patients aged <18 years after 24 and 36 months was −0.94 (0.95; n = 10) and −0.73 (1.41; n = 4), respectively. No new safety issues were observed during long-term treatment.

Setmelanotide demonstrated clinical benefit on body weight with up to 3 years of treatment. These data support the long-term use of setmelanotide for patients with POMC, PCSK1, and LEPR biallelic deficiency.

Disclosures: None

### P53 Body Mass Index and Weight Reductions in Patients With Obesity Due to Heterozygous Variants in POMC, PCSK1, and LEPR After 1 Year of Setmelanotide

#### Farooqi S^1^, Miller J^2^, Still C^3^, Scimia C^4^, Ohayan O^4^, Yuan G^4^, Yohn M^3^, Argente J^5,6^

##### ^1^University of Cambridge, ^2^University of Florida, USA,^4^Rhythm Pharmaceuticals, USA, ^5,6^Universidad Autónoma de Madrid, Spain

Heterozygous variants in genes upstream of the melanocortin-4 receptor (MC4R) including POMC, PCSK1, and LEPR can cause hyperphagia and early-onset, severe obesity. The MC4R agonist setmelanotide reduced weight and hunger after 3 months in a Phase 2 trial of patients with obesity due to these heterozygous variants. We assessed the continued efficacy of setmelanotide after a year of treatment in these patients.

Patients aged ≥6 years with obesity due to heterozygous POMC, PCSK1, and LEPR variants began this long-term extension (LTE) trial (NCT03651765) after completing an index trial where they received ≥4 months of setmelanotide and demonstrated clinical benefit. Study visits occurred every 3 months and evaluated safety, tolerability, and changes in weight-related measures. This analysis reports outcomes after 1 year of setmelanotide across the index and LTE trials.

As of October 29, 2021, 35 eligible patients had enrolled in the index trial. Sixteen, 17, and 17 patients continued into the LTE and received ≥6, 9, and 12 months of setmelanotide, respectively. At index trial baseline, mean (standard deviation [SD]) body mass index (BMI) was 50.26 (9.41) kg/m2. Mean (SD) percent change in BMI was −6.93% (9.13%; n = 16), −8.14% (10.13%; n = 17), and −7.83% (9.69%; n = 17) after 6, 9, and 12 months of treatment, respectively. Of patients ≥18 years old, the mean (SD) percent change in weight was −10.24% (7.90%; n = 15) after 12 months. For 1 patient <18 years old, change in BMI Z score was 0.64 after 12 months. No new safety concerns emerged during the LTE; 1 patient discontinued because of an adverse event, unrelated to treatment.

Setmelanotide demonstrated efficacy in patients with obesity due to heterozygous variants in POMC, PCSK1, and LEPR after 1 year of treatment, supporting the continued investigation of setmelanotide in these patients, underway in the Phase 3 EMANATE trial (NCT05093634).

Disclosures: None

### P54 Long-term Efficacy of Setmelanotide in Patients With Bardet-Biedl Syndrome

#### Argente J^1,2^, Haqq A^3^, Clément K^4,5^, Chung W^6^, Dollfus H^7^, Forsythe E^8^, Beales P^8^, Martos-Moreno G^1^, Yanovski J^9^, Mittleman R^10^, Yuan G^10^, Haws R^11^

##### ^1 2^IMDEA Food Institute Spain, ^3^University of Alberta Canada, ^4,5^Pitié-Salpêtrière Hospital, France, ^6^Columbia University USA, ^7^Hôpitaux de Strasbourg, France, ^8^GOSH, UK, ^9^NIH, USA,^10^Rhythm Pharmaceuticals USA, ^11^Marshfield Clinic Research USA

Bardet-Biedl syndrome (BBS) is a rare genetic disease characterized by hyperphagia and early-onset, severe obesity believed to be driven by impaired signaling in the melanocortin-4 receptor (MC4R) pathway. In Phase 2 and 3 trials, treatment with the MC4R agonist setmelanotide reduced weight, body mass index (BMI), BMI Z score, and hunger in patients with BBS at ~1 year. Here, we report results from a long-term extension (LTE) trial of setmelanotide (NCT03651765).

Methods: Patients with BBS aged ≥6 years were eligible if they demonstrated clinical benefit and acceptable safety following ~12 months of setmelanotide treatment in an index trial. Patients began the LTE immediately following index trial completion. Study visits occurred every ~3 months in the LTE. Outcomes were assessed relative to the index trial baseline.

Results: Of patients in the LTE, 30 and 19 received at least 18 and 24 months of treatment, respectively. At the index trial, baseline mean (standard deviation [SD]) BMI was 42.2 (9.2) kg/m2, body weight in patients ≥18 years was 132.3 (20.9) kg, and BMI Z score in patients <18 years was 3.5 (0.76). Across age groups, after 18 and 24 months of treatment, mean (SD) percent change in BMI was −9.5% (10.5%; n = 30) and −14.3% (11.6%; n = 19) respectively. Mean (SD) percent change in body weight in those ≥18 years after 18 and 24 months was −8.6% (10.3%; n = 15) and −14.9% (10.4%; n = 6), respectively. Mean (SD) change in BMI Z score in patients <18 years after 18 and 24 months was −0.83 (0.50; n = 13) and −0.72 (0.54; n = 12), respectively. No new safety signals were observed. One patient discontinued during the LTE because of an adverse event unlikely related to setmelanotide (auditory hallucination).

Setmelanotide continued to have clinical benefit and was generally well tolerated, supporting longer-term use of setmelanotide in BBS.

Disclosures: None

### P55 Protocol for a multi-centre intervention to study short and medium-term effects of sweeteners on appetite-related behaviour, physiology, and health

#### O’Hara D, O’Connor D, Beaulieu K, Almiron-Roig K, Martinez JA, Halford JCG,

##### Harrold J, Raben A, Hardman C, Nazarre J-A, Finlayson G, Gibbons C on behalf of the SWEET Consortium University of Leeds

The replacement of free sugars with low/zero energy sweeteners and sweetness enhancers (S&SEs) in food products is one method to reduce sugar intake while maintaining acceptance and palatability of the diet. As a result of consumer demand and various health policies (e.g., â€˜sugar taxesâ€™) the presence of S&SEs in the food supply is increasing. However, there is controversy surrounding the short- and medium-term impact of S&SE-containing products on appetite, and little is known about the mechanisms of action of S&SE types or blends. This multi-centre, double blind, cross-over, RCT aims to evaluate the effect of acute and repeated consumption of novel S&SE blends in semi-solid and solid foods on appetite and related behavioural, metabolic and health outcomes. Five trials will be carried out at 5 intervention sites across 4 countries including 213 individuals with overweight or obesity. Five food matrices (cake, biscuits, yoghurt, chocolate, and breakfast cereal) will be tested across three formulations (sucrose-sweetened control versus two reformulated products with S&SE blends) over a 14-day period. The primary endpoint is composite appetite score using 3-hour postprandial incremental area under the curve (iAUC) after 2 weeks. Secondary endpoints include food reward, food cravings, satiety biomarkers, hepatic function and glycaemic markers, and gastro-intestinal symptoms.

Disclosures: JCGH, JAH and CH and are in receipt of research funding from the American Beverage Association. CH has received honoraria from the International Sweeteners Association. AR has received honoraria from Unilever and the International Sweeteners Association.

### P56 HABIT (Health After Birth Intervention Trial) A feasibility randomised controlled trial investigating the effect of a kilocalorie controlled, low carbohydrate dietary intervention, behavioural modification and telehealth versus a standard 1:1 NHS weight management programme in post-partum women living with obesity

#### Graham A, Smith S, Baer G, Reynolds, R

##### Queen Margaret University

Elevated maternal body mass index is associated with an array of adverse health outcomes. The postpartum phase is considered an optimal time for weight management however, with little advancement in identifying effective weight management interventions postpartum, further research is warranted. HABIT aims to assess the feasibility of study interventions and utilise derived data to inform the feasibility of a larger, definitive trial. The study will determine the effectiveness of a standard NHS 1:1 weight management programme versus an adapted 1:1 programme encompassing a low carbohydrate dietary intervention, telehealth, enhanced behaviour change and increased self-monitoring.

HABIT is a UK based, parallel, non-blinded, feasibility randomised controlled trial. Eligible participants will have a booking BMI of ≥30kg/m^2^, recently given birth or about to give birth and meet the study inclusion criteria. Participants may be recruited by Midwives, Health Visitors, Dietitians within a gestational diabetes mellitus workstream, clinicians within a metabolic antenatal clinic and via social media. Participants will be randomly assigned (1:1) to receive a 12-week standard NHS 1:1 weight management programme, advocating a low fat, high carbohydrate dietary approach (active control) or an adapted 1:1 programme comprised of a low carbohydrate dietary intervention defined as 50-130g daily carbohydrate (experimental). The experimental group will receive a personalised daily kilocalorie and step count target. The telehealth system Florence will prompt self-recording of daily pedometer readings, weekly weight and deliver weekly enhanced behaviour change messages. The primary outcome is bodyweight. Secondary outcomes include body composition, waist circumference, blood pressure, physical activity level, quality of life, psychological distress, hunger and eating cues, emotional eating, nutritional adequacy and food consumption trends.

Disclosures: None

### P57 Utilising the planning system to restrict the accessibility of hot food takeaways: barriers and facilitators

#### O’Malley C, Lake AA, Bradford C, Townshend T, Gray N, Moore H

##### Teesside University, Newcastle University, Fuse: The Centre for Translational Research in Public Health

Healthier food environments encourage individuals to make better food choices more frequently, reducing ill health and obesity. The planning system can be utilised to promote healthier food environments by controlling the accessibility of hot food takeaways (HFTs). New applications for HFTs can be rejected by local authorities (LAs) under certain circumstances, however, these decisions are subject to appeal; where they are either upheld or dismissed by the National Planning Inspectorate. The aim of this research was to explore the barriers and facilitators to implementing this HFT-restricting mechanism at local and national levels.

Forty-seven HFT appeals cases were identified from the Appeals Database in England between December 2016 and March 2020. A mapping exercise identified eight recent case study sites where a new HFT was either upheld (n = 4) or dismissed (n = 4) after appeal. For each site, documents applicable to each case were analysed and a total of 11 professionals, 1 member of the public, and 1 representative from the Planning Inspectorate were interviewed. A survey was also completed by 7 LA health professionals. Three interpretation meetings were conducted with participants and a wider group of stakeholders to clarify and sense check findings.

Participants perceived that LAs would be better equipped to defend HFT appeals if professionals had (1) a greater understanding of applying Local Planning Policy and Supplementary Planning Documents correctly. (2) Adequate staffing capacity for dealing with appeals cases. (3) Access to accurate, robust, and current information that is interpretable across professional groups. (4) Support and commitment from elected members and senior management. (5) Good lines of communication with relevant local groups and communities interested in the appeal.

Communication across professional groups appeared to be a key factor in successfully defending takeaway appeal cases, to facilitate the sharing of, and signposting to, useful information.

Disclosures: None

### P58 Influences of school catering contracts on the nutritional adequacy of food offered to children in UK schools

#### Blacklock A, Lake AA, Moore HJ, O’Malley CL, Tennyson C, Gray N

##### Teesside University; Redcar and Eston School Sports Partnership

In England, school food standards were abandoned in the 1980â€™s which led to a decline in the quality of food offered to children within schools. In 2008 food-based standards were introduced, although still contributed little to a healthy balanced diet. From 2014, the UK government imposed stricter school food standards in an attempt to improve the state of the diets of the nationâ€™s youth. As a child will obtain one third of their daily diet from school, the impact of school meals cannot be underestimated, particularly when an increasing number of children do not have access to a hot, nutritious meal at home.

Using questionnaires, interviews and Braun and Clarke reflexive thematic analysis, this study assessed the influence the school food contract has on the catering possibilities within Multi Academy Trusts by exploring who is responsible for the contract and the processes followed for quality assurance. These results were used alongside academic literature and government guidance to cross analyse potential barriers for school food catering services and identify potential improvements and changes.

The data suggests that a select few senior school figures and the governing body have overall authority regarding the school catering contract and that there is a lack of standardised quality assurance processes across schools and trusts resulting in vast disparities in the quality of food offered.

Via real-world accounts and literature analysis, this research has identified six key recommendations to facilitate dietary equity and accessibility for all children within the UK; whole school approach, standardised school food monitoring system, parental communication, staff autonomy, full school access to free school meals, schools being allowed to prohibit inappropriate foods.

Disclosures: None

### P59 Food insecure women’s experiences of their nutritional health and wellbeing in Europe: a qualitative systematic review and meta-ethnography

#### Bell Z, Scott S, Visram S, Rankin J, Bambra C, Heslehurst N

##### Newcastle University

There has been a rise in food insecurity since the 2008 global financial crisis. Particularly vulnerable are mothers with young children, pregnant women, and lone parents (the majority of whom are women). This systematic review and meta-ethnography of qualitative studies aimed to explore women’s experiences of food insecurity and how it affects their nutritional health and wellbeing.

Six electronic databases were searched from 01/01/2008-10/07/2021, supplemented by grey literature, reference list and citation searches. Screening was performed in duplicate. Following title and abstract screening, full texts were screened against the inclusion criteria (qualitative research, focus on women of childbearing age/pregnancy nutritional health/well-being, data from 2008 onwards, high-income European countries). We adhered to PRISMA and eMERGe guidelines for meta-ethnographic reporting. Methodological quality was assessed using the CASP qualitative checklist. Data were synthesised according to Noblit & Hare’s seven phases of meta-ethnography.

We identified 11,589 unique records; 23 publications reporting data from 22 unique studies were included, comprising 647 women. We identified two key themes: accessing sufficient food and embodying food insecurity, comprising eight sub-themes. Our meta-ethnography provides a progressive storyline of women’s experiences of food insecurity. This includes how women attempt to access sufficient food, they are unable to meet their nutritional needs, how this is embedded into their everyday lives and embodied in unhealthful physical, social, and mental nutritional health and wellbeing.

This meta-ethnography concludes that there needs to be greater recognition of the psychosocial impact of food insecurity on vulnerable women, in addition to the impact on their nutritional health and wellbeing. A strength and limitation of this review is the diverse range of included studies, from different European contexts, where contextual factors could impact the experiences of women.

Disclosures: None

### P60 Improving the nutritional follow-up for bariatric patients in a primary care setting

#### Kim WC, Im CR

##### Oxford University Hospital Foundation Trust

Bariatric surgery is currently one of the most effective management options for patients with morbid obesity, aiming to reduce the risks of multiple comorbidities and improve cost-efficiency. With effective treatment, many health improvements are seen, including but not limited to a reduction of cardiovascular disease, type 2 diabetes mellitus, and all-cause mortality, whilst inadequate follow-up can lead to the development of weight regain and nutritional deficiencies. The British Obesity & Metabolic Surgery Society (BOMSS) have published recommended guidelines for post-operative care and monitoring upon discharge from the bariatric centre.

A general practice database in Oxford was searched to identify patients registered as active members of the practice with a history of bariatric surgery. Data on the patient demographics, which surgical procedure they received (laparoscopic adjustable gastric bypass (LAGB), Roux-en-Y bypass, or sleeve gastrectomy), and what follow-up tests and prescriptions they were receiving in line with the BOMSS guidelines were collated.

9 patients in total were identified, 4 with a LAGB, 3 with a sleeve gastrectomy, and 2 with a Roux-en-Y bypass. None of the patients had been seen 1 year after discharge from the bariatric service by a general practitioner. 4 of the 5 patients eligible for B12 injections were regularly receiving treatment, and 2 of those patients were receiving regular blood tests alongside. 5 of the patients were receiving repeat dispensing prescriptions of calcium and/or multivitamins.

There is currently a lack of adequate follow-up upon discharge from the bariatric service. Education and reminders of how and what follow-up is required should be provided for all members of the practice, as well as clear communication between the bariatric and primary care service teams, to provide a better flow of care for bariatric patients and prevent further complications.

Disclosures: None

### P61 Planning, Hot Food Takeaways & COVID-19: perceptions of the impact of regulations on access to healthy food environments

#### Bradford C, O’Malley C, Moore H, Chang M, Mathews C, Townshend T, Gray N, Lake AA

##### Teesside University / Newcastle University / Department of Health and Social Care

Planning regulations can be used to improve food environments by preventing the over-proliferation of hot food takeaways (HFTs). In March 2020 the UK Government introduced temporary changes in planning regulations across England to help mitigate the effects of lockdown. These changes allowed food retailers to trade as takeaway services without needing to apply for planning permission. The aim of this research was to better understand the impact of these regulations on relevant professional groups.

Between January to March 2021, a focus group and interviews were conducted with 15 professionals across seven of the 12 North-East local authorities (LAs). Professionals included Planners, Public Health Leads, Environmental Health Officers, and Town Centre Managers. A year later, (March 2022), follow-up interviews and focus groups were conducted with a sample of North-East based professionals (n = 16), (including several from the original cohort) to further understand how the situation had developed. Data was analysed using a codebook thematic analysis approach.

General consensus was that most businesses who utilised temporary regulations did not notify their LA, despite it being a legal requirement. LAs were unaware of any formal data collection taking place, and one year on the role of collecting this data still appeared to be unassigned, with services continuing to play catch-up over time lost due to Covid-19. Professionals were concerned about the potential long-term health consequences (especially obesity-related outcomes) and the challenges of reversing the uptake of the regulations, including a sense that businesses may ignore the end of the regulations if they are not actively enforced.

The food environment is constantly evolving; with the recent increase in ‘dark kitchens’ and online delivery services (e.g., Deliveroo), the ending of the regulations may have been partially nullified, as the line which defines a takeaway from a restaurant becomes increasingly blurred.

Disclosures: None

### P62 The effects of transcranial direct current stimulation (tDCS) on food craving, food reward, and subjective appetite in those with binge-type eating behaviour

#### Beaumont JD, Dalton M, Davis D, Finlayson G, Russell M, Barwood MJ

##### Leeds Trinity University, University of Leeds

Transcranial direct current stimulation (tDCS) has been shown to attenuate reward response to food, reducing food cravings and consumption. Those displaying eating behaviour traits suggesting susceptibility to overconsumption (e.g., binge eating behaviour) appear most responsive to the modulatory influence of tDCS. The present work explored the impact of tDCS in those with subclinical binge-type behaviour, and whether an eating behaviour trait-dependent effect is present.

Seventeen female participants (23 Â ± 7 years, 25.4 Â ± 3.8 kgÂ·cmâˆ’2) displaying mild-to-moderate binge eating behaviour completed two sessions of double-blind, randomised and counterbalanced active or sham tDCS over the dorsolateral prefrontal cortex at 2 milliampere for 20 minutes. Subjective appetite visual analogue scales, the Food Craving Questionnaire-State, and Leeds Food Preference Questionnaire were completed at baseline, immediately post-tDCS and after a standardised meal (cheese sandwich providing 30% resting metabolic rate). Data were compared using analysis of variance to 0.05 alpha level.

Baseline hunger was significantly different between conditions (p = 0.035). There were no difference in pre- and post-tDCS scores across subjective appetite and food craving measures when comparing tDCS conditions (p â‰¥ 0.127). Explicit liking (p = 0.016) and wanting for high-fat sweet foods (p = 0.008) were significantly different, with increased scores following active tDCS. These differences were eradicated when controlling for baseline hunger (liking: p = 0.161; wanting: p = 0.138). Inclusion of post-meal scores in analyses showed a similar pattern of effects.

Our data may suggest the eating behaviour traits displayed by participants were below the threshold required to be responsive to tDCS. Prior work suggests clinically-relevant binge eating behaviour results in modulation of eating behaviour through tDCS, indicating subclinical populations may be unresponsive to tDCS. Future work should directly compare the effects in clinical and sub-clinical populations displaying eating behaviour traits susceptibility to overconsumption.

Disclosures: None

### P63 Neurobehavioral markers of food preference and reward in fasted and fed states and their association with eating behaviors in young Chinese adults

#### Zhou Y, Finlayson G, Zhou C

##### University of Leeds

Adverse neurobehavioral responses to food could be one of the contributing factors to obesity in China, which is a major public health issue. There is a need for methodologies to better understand the cognitive processes and neural mechanisms underlying eating behaviors. This study investigated neurobehavioral markers of food preference and reward in pre- and post-meal states, and their association with eating behaviors in young Chinese adults. Chinese adults (N = 80: 39 male) completed the Leeds Food Preference Questionnaire (LFPQ-CH), and a Functional Near-Infrared Spectroscopy (fNIRS) Visual Food Cues Paradigm before and after an ad libitum test meal. The Dutch Eating Behavior Questionnaire (DEBQ) and VAS appetite scores were used to measure appetitive traits and states. Results showed that categorisation of foods along dimensions of sweetness and fat content were valid for the general population in China. Test meal-induced changes in food preferences and increased dorsolateral prefrontal cortex (DLPFC) activation to high-fat savoury (HFSA) food were demonstrated. Regression analyses using LFPQ-CH and fNIRS variables as predictors of ad libitum food intake and DEBQ traits showed that variance in these eating behaviours were significantly associated with LFPQ-CH scores, and fNIRS activity could explain additional unique variance in these outcomes. The current results support the use of LFPQ-CH with fNIRS as valid and complementary tools for the neurobehavioral evaluation of food reward and preference. This methodological approach is sensitive to changes in appetitive state; can differentiate along the spectrum of eating behaviour traits and is predictive of food intake.

Disclosures: None

### P64 Latent Profile Analysis of Children’s Eating Behaviour: Identifying Avid Eaters

#### Pickard A, Farrow C, Llewellyn C, Haycraft E, Croker H, Herle M, Blissett J

##### Aston University

Previous research has evidenced that from infancy onwards genetic influence on weight is partly mediated by appetite avidity, expressed as food approach (FA) behaviours. Children who are more food responsive eat more frequently throughout the day than children who are less responsive to food cues, increasing susceptibility to future obesity. However, no research has yet identified a distinct eating behaviour profile reflecting high food FA/appetite avidity in children, or examined how other factors such as child temperament, experience of food insecurity, or parental feeding practices, may vary by that profile.

An online survey was conducted with 995 parents/carers living in England and Wales. Participants reported on their child’s eating behaviour using the Child Eating Behaviour Questionnaire (CEBQ), as well as completing measures of child’s temperament (VSF-CBQ), household food security (HFSS) and parental feeding practices (CFPQ). Latent Profile Analysis (LPA) was carried out to identify distinct eating profiles amongst the children (36-72 months, Mage = 48.8 months, 52% female).

Three profiles emerged: (a) avid eaters, (b) typical eaters, (c) fussy eaters. Avid eaters (32.6% of children) were characterised by higher food responsiveness, enjoyment of food, and emotional over-eating in combination with lower satiety responsiveness, slowness in eating, emotional under-eating, and food fussiness. Overall, the findings suggest distinct eating behaviour profiles, which differ in child temperament and parent feeding practice. Avid eaters are reported to be more surgent, with less display of negative emotion, and are the recipients of a range of adaptive feeding practices, including monitoring, but are subject to greater parental control over their eating. There was no significant difference in food insecurity between the three eating profiles. Further work to establish the causal relationships at play are necessary to understand how to protect avid eaters from overweight and less healthy eating.

Disclosures: None

### P65 The moderating and mediating role of eating behaviour traits in acceptance and commitment therapy based weight management interventions: Protocol for an individual participant data meta-analysis

#### Kudlek L, Eustachio Colombo P, Mueller J, Ahern A

##### University of Cambridge

Precision medicine approaches to obesity aim to maximise treatment effectiveness by matching weight management interventions (WMIs) to individual characteristics that influence outcomes. Eating behaviour traits (EBTs), such as uncontrolled eating and emotional eating, may be useful targets. Initial evidence suggests that Acceptance and Commitment Therapy (ACT) - based WMIs might address these EBTs more effectively than standard behavioural approaches and might be most effective in people with high levels of these traits. However, few studies have examined this directly. This systematic review and Individual Participant Data (IPD) meta-analysis will examine (1) whether ACT-based WMIs are more effective for people with certain levels of EBTs and (2) whether ACT-based WMIs operate through changes in EBTs.

Eligible studies that evaluated ACT-based WMIs for people with BMI > 25 and assessed EBTs at baseline or follow-up were identified by screening studies from a previous review (Lawlor et al., 2019) and updating the search to 20.06.2022. IPD will be requested from all eligible published and unpublished studies and we will contact study authors when eligibility is unclear. Once received, IPD will be harmonised, and studies will be re-analysed in a two-stage IPD meta-analysis to examine whether (1) baseline EBTs moderate the effect of ACT-based WMIs on weight and (2) changes in EBTs mediate the effect of ACT-based WMIs on weight. Where authors are unable or unwilling to share IPD, we will offer them the opportunity to conduct the specified analyses and share the output instead of IPD to be incorporated in the meta-analysis. Risk of bias of included trials will be assessed using the Cochrane Risk of Bias tool 2 (RoB2) and the Risk of Bias in Non-randomized Studies of Interventions tool (ROBINS-I).

Findings will be published in peer-reviewed journal and will contribute to the lead author’s thesis.

Disclosures: None

### P66 Comprehensive application of a systems approach to obesity prevention: a scoping review of empirical evidence

#### Bai L, Mohammed A, Steve A, Boyd S, Remco P, Charlie F

##### University of Bristol

A systems approach to obesity prevention is increasingly urged. However, confusion exists on what a systems approach entails in practice, and the empirical evidence on this new approach is unclear. This scoping review aimed to identify and synthesise studies/programmes that have comprehensively applied a systems approach to obesity prevention. By searching international databases and grey literature, five publications (three studies) met the inclusion criteria. The review also showed that misunderstanding of the systems approach and poor reporting might partly explain why only a small number of studies met the inclusion criteria. No conclusion on the effectiveness of this approach can be drawn yet due to the extremely limited evidence base. We identified common features shared by the included studies, such as measuring ongoing changes, in addition to endpoint outcomes and supporting capacity building. Some facilitators and barriers to applying a comprehensive systems approach in practice were identified. Facilitators include using Group Model Building workshops and focusing on community assets rather than needs or lacks. A further facilitator was related to triggers to personal involvement in the programme and perceived prompts for others to participate. On the other hand, the identified barriers were related to the miscommunication and confusion observed within the steering group organisation and the unforeseen social and economic shocks. A further obstacle was related to the standard processes of Group Model Building workshops that were not adapted to support community members with low health literacy. More well-designed and reported studies are needed, especially from low- and middle-income settings.

Disclosures: None

